# Regionales Monitoring von Infektionen mittels standardisierter Fallfatalitätsraten am Beispiel von SARS-CoV-2 in Bayern

**DOI:** 10.1007/s00103-021-03397-8

**Published:** 2021-08-12

**Authors:** Kirsi Manz, Ulrich Mansmann

**Affiliations:** 1 Institut für Medizinische Informationsverarbeitung, Biometrie und Epidemiologie (IBE), Ludwig-Maximilians-Universität München, Marchioninistr. 15, 81377 München, Deutschland; 2 Pettenkofer School of Public Health (PSPH), Ludwig-Maximilians-Universität München, München, Deutschland

**Keywords:** Geographische Epidemiologie, Bayesianische hierarchische Modelle, Indirekte Standardisierung, Standardisierte Mortalitätsrate, Standardisierte Inzidenzrate, Geographical epidemiology, Bayesian hierarchical models, Indirect standardization, Standardized mortality rate, Standardized incidence rate

## Abstract

**Hintergrund:**

Karten zur zeitlichen Entwicklung der regionalen Verteilung einer gesundheitsbezogenen Maßzahl ermöglichen public-health-relevante Bewertungen des Gesundheitsgeschehens.

**Ziel der Arbeit:**

Die Arbeit führt das Konzept der standardisierten Fallfatalitätsrate (sFFR) ein. Sie beschreibt das Verhältnis der regionalen Abweichung in der Mortalität zur regionalen Abweichung im dokumentierten Infektionsprozess. Die regionalen sFFR-Werte werden in Karten dargestellt und die darin beobachtete zeitlich variierende regionale Heterogenität interpretiert.

**Materialien und Methoden:**

Die regionale sFFR ist der Quotient der regionalen standardisierten Mortalitäts- und Fallrate. Sie wird mittels eines bivariaten Modells geschätzt. Die in Karten dargestellten sFFR-Werte basieren auf den SARS-CoV-2-Meldedaten aus Bayern seit Anfang April 2020 bis Ende März 2021. Es werden 4 Quartale (Q2/20, Q3/20, Q4/20, Q1/21) betrachtet.

**Ergebnisse:**

In den betrachteten Quartalen liegen die bayerischen naiven FFR-Werte bei 5,0 %, 0,5 %, 2,5 % und 2,8 %. In Q2/20 sind die regionalen sFFR-Werte unregelmäßig über das Bundesland verteilt. Diese Heterogenität schwächt sich in der zweiten Welle der Epidemie ab. In Q1/21 zeigen sich in Südbayern nur vereinzelt Regionen mit erhöhter sFFR (> 1,25). Im Norden Bayerns bilden sich Cluster von Regionen mit einer sFFR > 1,25. Das Oberallgäu ist die Region mit dem niedrigsten sFFR-Wert (0,39, 95 % Kredibilitätsintervall: 0,25–0,55).

**Diskussion:**

In Bayern zeigen sich heterogene regionale SARS-CoV-2-spezifische sFFR-Werte, die sich über die Zeit verändern. Sie schätzen das relative Risiko, als dokumentierter Infektionsfall an/mit COVID-19 zu versterben. Eine starke kleinräumige Variabilität der sFFR legt nahe, regionale gegenüber übergeordneten Maßnahmen zur Steuerung des Infektionsgeschehens vorzuziehen.

**Zusatzmaterial online:**

Zusätzliche Informationen sind in der Online-Version dieses Artikels (10.1007/s00103-021-03397-8) enthalten.

## Abstract (en)

**Background:**

Maps of the temporal evolution of the regional distribution of a health-related measure enable public health-relevant assessments of health outcomes.

**Objectives:**

The paper introduces the concept of standardized case fatality rate (sCFR). It describes the ratio of the regional variation in mortality to the regional variation in the documented infection process. The regional sCFR values are presented in maps and the time-varying regional heterogeneity observed in them is interpreted.

**Materials and methods:**

The regional sCFR is the quotient of the regional standardized mortality and case rate. It is estimated using a bivariate model. The sCFR values presented in maps are based on SARS-CoV‑2 reporting data from Bavaria since the beginning of April 2020 until the end of March 2021. Four quarters (Q2/20, Q3/20, Q4/20, and Q1/21) are considered.

**Results:**

In the quarters considered, the naïve CFR values in Bavaria are 5.0%, 0.5%, 2.5%, and 2.8%. In Q2/20, regional sCFR values are irregularly distributed across the state. This heterogeneity weakens in the second wave of the epidemic. In Q1/21, only isolated regions with elevated sCFR (> 1.25) appear in southern Bavaria. Clusters of regions with sCFR > 1.25 form in northern Bavaria, with Oberallgäu being the region with the lowest sCFR (0.39, 95% credibility interval: 0.25–0.55).

**Conclusions:**

In Bavaria, heterogeneous regional SARS-CoV-2-specific sCFR values are shown to change over time. They estimate the relative risk of dying from or with COVID-19 as a documented case. Strong small-scale variability in sCFR suggests a preference for regional over higher-level measures to manage the incidence of infection.

## Einleitung

Indikatoren zum Infektionsgeschehen wie zum infektionsbedingten Versterben liefern in einer Epidemie wichtige Informationen zur Bewertung der Gesundheitslage wie auch zur Steuerung von Maßnahmen zur Kontrolle der Epidemie. Fallfatalitätsraten werden regional aufgelöst nicht kommuniziert. Zu diesem Zweck werden Inzidenz- und Mortalitätsraten simultan berichtet. Die folgende Arbeit führt das Konzept der standardisierten Fallfatalitätsrate (sFFR) ein. Die sFFR erlaubt das Monitoring des relativen Risikos, als dokumentierter Fall an/mit COVID-19 zu versterben.

Die *regionale standardisierte COVID-19-Letalität* (standardisierte Fallfatalitätsrate, sFFR_r_) quantifiziert die relative Abweichung der naiven regionalen *Fallfatalitätsrate* (nFFR_r_) zur populationsbasierten nach dem übergeordneten Standard zum Infektionsgeschehen *erwarteten Fallfatalitätsrate* (eFFR_r_): sFFR_r_ = nFFR_r_ / eFFR_r_. Die sFFR_r_ quantifiziert infektionsbezogene Über- oder Untersterblichkeit bezüglich eines Standards. In ihre Berechnung gehen die Fallsterblichkeit und ein überregionaler nach Alter und Geschlecht stratifizierter Populationsstandard zum Infektionsgeschehen und Versterben der dokumentierten Infizierten ein.

Tab. 1 verdeutlicht die Berechnung der sFFR_r_ an 2 Regionen mit gleichen Einwohnerzahlen, gleichen absoluten Infektionszahlen und infektionsbedingten Todesfällen. Die Regionen unterscheiden sich in ihrer Altersstruktur. Die Population ist in 3 Altersgruppen (A1, A2, A3) stratifiziert, worauf sich auch der übergeordnete Standard und die beobachteten Daten beziehen. Region 1 hat eine größere naive (nFFR_1_ = 0,292) im Vergleich zu der erwarteten FFR (eFFR_1_ = 0,216). Die sFFR für Region 1 ist sFFR_1_ = 1,35. Region 2 hat die gleiche naive FFR wie Region 1 (nFFR_2_ = 0,292). Diese ist kleiner als die bei gegebener Populationsstruktur erwartete FFR (eFFR_2_ = 0,393). Die sFFR für Region 2 ist somit sFFR_2_ = 0,74. Trotz gleicher Populationsgröße, gleicher dokumentierter Infektionszahlen und infektionsbedingter Sterbefälle ergibt sich eine zum Standard geringere infektionsbedingte Sterblichkeit in Region 2 als in Region 1. Weitere Beispiele finden sich im Onlinematerial zu diesem Beitrag in Tabelle Z1.

**Table 1:** 

**Standard**			
*Gruppe*	*Infektionen*	*Infektionsbedingte Todesfälle*	*Pro XXX Einwohner*
*A1*	50	1	1000
*A2*	100	10	1000
*A3*	150	75	1000
**Region 1**			
*Gruppe*	*Infektionen*	*Infektionsbedingte Todesfälle*	*Einwohnerzahl*
A1	1000	20	20.000
A2	5000	400	20.000
A3	4000	2500	10.000
*nFFR*_*1*_	0,292	*sFR*_*1*_	2,222
*eFFR*_*1*_	0,216	*sMR*_*1*_	3,010
*sFFR*_*1*_	1,355	*sFFR*_*1*_	1,355
**Region 2**			
*Gruppe*	*Infektionen*	*Infektionsbedingte Todesfälle*	*Einwohnerzahl*
A1	1000	20	10.000
A2	5000	400	10.000
A3	4000	2500	30.000
*nFFR*_*2*_	0,292	*sFR*_*2*_	1,667
*eFFR*_*2*_	0,393	*sMR*_*2*_	1,237
*sFFR*_*2*_	0,742	*sFFR*_*2*_	0,742

*A* Altersgruppe, *nFFR* naive Fallfatalitätsrate, *eFFR* erwartete FFR, *sFFR* standardisierte FFR, *sFR* standardisierte Fallrate, *sMR* standardisierte Mortalitätsrate

Ein starker regionaler Infektionsausbruch oder verstärktes regionales Testen auf SARS-CoV‑2 verringern die sFFR_r_. In beiden Szenarien erhöht sich die Infektionsinzidenz und reduziert bei gleichbleibenden Sterbezahlen die regionale sFFR_r_. Schulöffnungen können ähnlich wirken. Kommt es andererseits in einer Region durch eine starke Zunahme der Infektionszahlen zu Engpässen in der Versorgung der schwer an COVID-19 erkrankten Personen, erhöht dies die sFFR_r_. Die sFFR_r_-Werte reflektieren somit Aspekte der Versorgung wie auch der Infektionsprävention.

Hieraus ergibt sich ein erster Interpretationsversuch für die sFFR_r_: Werte größer 1 zeigen Situationen mit einem höheren regionalen Sterberisiko für Infizierte verglichen mit dem Standard. Werte größer 1 weisen auf Maßnahmen zur Reduktion der Sterblichkeit bei Infizierten hin (etwa durch Veränderung in Versorgungsstrukturen). Werte deutlich kleiner 1 deuten auf ein geringeres Sterberisiko für Infizierte und implizieren eine Konzentration auf die Reduktion des Infektionsrisikos (Priorisierung von Kontaktbeschränkungen). Starke kleinräumige Variabilität der sFFR_r_-Werte legt nahe, zur Steuerung des Infektionsgeschehens regionale gegenüber übergeordneten Maßnahmen vorzuziehen.

Die Vorbehalte dieses Interpretationsversuchs werden an praktischen und theoretischen Beispielen diskutiert. Die Berechnung der sFFR_r_ mithilfe indirekter Standardisierung ist ebenfalls bei der Interpretation zu berücksichtigen.

Karten stellen relevante regionale Gesundheitsstatistiken auf Flächen (Regionen in einem übergeordneten Bereich) dar, die bestimmte Werte in unterschiedlichen Farben anzeigen. Komplexe raumzeitliche Prozesse in der Dynamik der Epidemie werden durch das Nebeneinanderstellen der sFFR_r_-basierten Karten über einen Zeitverlauf sichtbar. Erlaubt die zeitliche Beobachtung der regionalen sFFR_r_ einen neuen Blick auf die Dynamik der Infektion? Wie verhalten sich sFFR_r_-Werte während der bisher beobachteten beiden Infektionswellen?

Dieser Artikel präsentiert sFFR_r_-Karten für die 96 bayerischen Landkreise und kreisfreien Städte (Regionen) über die 4 Quartale Q2/20, Q3/20, Q4/20, Q1/21 (01.04.2020 bis 31.03.2021). Die Karten vergleichen die Regionen zu einem geschlechts- und altersstratifizierten Standard. Der Standard kann extern definiert sein (Europa, eine andere Nation, ein anderes Bundesland): Wie steht Bayern bei einem entsprechenden nationalen oder internationalen Vergleich da? Für die in dieser Arbeit dargestellten Karten wird ein interner Standard gewählt: das Infektionsgeschehen in Bayern während der betrachteten Zeitperiode. Damit kann die bayernweite Heterogenität des Infektionsgeschehens untersucht werden: Wie variiert das standardisierte Sterberisiko von Infizierten? In welchen Regionen überwiegen standardisierte Sterbefallzahlen besonders stark im Vergleich zu standardisierten Infektionszahlen? Wo dominiert das Infektionsgeschehen bei guter Kontrolle der Sterblichkeit?

Hierarchische bayesianische Modelle zum *Disease Mapping* [1, 2] werden als formaler Rahmen für die statistische Behandlung der Daten gewählt. Sie erlauben eine Glättung der Karten und eine simultane Analyse der Infektions- wie auch Sterberisiken. Diese Verfahren erlauben die Quantifizierung der Unsicherheit (Kredibilitätsintervalle) der berechneten Maßzahlen und eine Adjustierung bezüglich regionaler Einflussgrößen.

Der Methodenteil beschreibt formale Eigenschaften der sFFR_r_ und den methodischen Rahmen für die Analyse der Daten. Der Ergebnisteil zeigt die erarbeiteten Karten. Die Diskussion beschäftigt sich mit der Interpretation der Ergebnisse und deren Vorbehalte und liefert eine kritische Bewertung des vorgelegten Konzeptes.

## Methoden

### Analyse der regionalen standardisierten FFR: sFFR_r_

Die Fallfatalitätsrate (FFR) beschreibt das Verhältnis der infektionsbezogenen Sterbefälle zur Anzahl der bekannten Infektionen.

Die erwartete Anzahl von Infektionsfällen pro geschlechtsspezifischer (i) Altersgruppe (j) und Zeitperiode (t) berechnet sich als die überregionale Inzidenzrate für dokumentierte Infektionen λ_t,i,j_^inf^ = I_t,i,j_ / pop_i,j_. Dabei ist I_t,i,j_ die für die Zeitperiode t überregional dokumentierte Anzahl von Infektionen in der jeweiligen geschlechtsspezifischen Altersgruppe und pop_i,j_ die entsprechende als zeitlich stabil angenommene überregionale Populationsgröße im demografischen Stratum (i,j). Für die erwarteten Infektionsfälle einer Region (r) multipliziert man pro Stratum die entsprechende überregionale Inzidenzrate mit der Bevölkerung in dem Landkreis (pop_r,i,j_) und summiert über die betrachteten Alters- und Geschlechtsgruppen: I_t,r_^erw^ = ∑_i,j_ λ_t,i,j_^inf^ × pop_r,i,j_. Analog dazu können die erwarteten Todesfälle pro Zeit und Region ermittelt werden: λ_t,i,j_^tod^ = T_t,i,j_ / pop_i,j_ wobei λ_t,i,j_^tod^ die überregionale Mortalitätsrate und T_t,i,j_ die Anzahl der an/mit der Infektion verstorbenen Personen ist. Danach erhält man T_t,r_^erw^ = ∑_i,j_ λ_t,i,j_^tod^ × pop_r,i,j_.

Das Verhältnis der beobachteten Todesfälle zu den beobachteten Infektionen ergibt die naive FFR einer Region r in einer Zeitperiode t:

$$\mathrm{nFFR}_{t,r}=T_{t,r}^{\mathrm{beob}}/I_{t,r}^{\mathrm{beob}}.$$

Das Verhältnis der erwarteten Todesfälle zu den erwarteten Infektionen ergibt die erwartete FFR einer Region r in einer Zeitperiode t:

$$\mathrm{eFFR}_{t,r}=T_{t,r}^{\mathrm{erw}}/I_{t,r}^{\mathrm{erw}}.$$

Für die standardisierte FFR gilt:

$$\mathrm{sFFR}_{t,r}=\mathrm{nFFR}_{t,r}/\mathrm{eFFR}_{t,r}=(T_{t,r}^{\mathrm{beob}}/I_{t,r}^{\mathrm{beob}})/(T_{t,r}^{\mathrm{erw}}/I_{t,r}^{\mathrm{erw}})=(T_{t,r}^{\mathrm{beob}}/T_{t,r}^{\mathrm{erw}})\times (I_{t,r}^{\mathrm{erw}}/I_{t,r}^{\mathrm{beob}})=\mathrm{sMR}_{t,r}/\mathrm{sFR}_{t,r}.$$

Dabei sind sMR_t,r_ und sFR_t,r_ die standardisierten Mortalitäts- und Fallraten pro Region und Zeitperiode. Sie sind relative Risiken (RR) zum Standard. Um Abweichungen in der sFFR besser zu verstehen, können die Effekte von Mortalität und Infektionsinzidenzen getrennt betrachtet werden. Es handelt sich also um ein Verhältnis relativer Risiken (Ratio of Relative Risks, RRR).

### Berechnung der geglätteten Karten für die standardisierten Raten (relative Risiken)

Kleinräumige Gesundheitsdaten zeigen Dateninstabilität. Die geografische Epidemiologie geht damit durch den Einsatz von Glättungsverfahren um und macht die Annahme, dass benachbarte Gebiete in ihren Eigenschaften ähnlicher als entferntere Gebiete sind [3]. Auf dieser Annahme aufbauende Methoden stabilisieren Schätzwerte, indem sie Informationen von benachbarten Gebieten („borrowing strength“) integrieren. Die Raten eines Gebietes werden zum Durchschnitt der Nachbargebiete und zum Landesdurchschnitt geglättet („shrinkage“). Je geringer die Bevölkerung des Gebietes, desto größer der Glättungseffekt. Dieses Vorgehen erhält alle räumlichen Informationen, minimiert zufällige Effekte und erzeugt räumliche Muster. Das Besag-York-Mollié-(BYM-)Modell [4] ist ein hierfür verwendetes Standardmodell. Es ist ein generalisiertes lineares Modell mit Poisson-Fehlerterm, das gebietsspezifische Zufallseffekte berücksichtigt. Eine Komponente modelliert die räumlich strukturierte Heterogenität (d. h. die Korrelationen zwischen benachbarten Gebieten), eine weitere die räumlich unstrukturierte Heterogenität. Da relative Risiken zu 2 simultanen Endpunkten betrachtet werden, wird die bivariate Version des BYM-Modells verwendet [2, 5].

Ein bayesianisches Modell verlangt Annahmen zu den notwendigen Aprioriverteilungen. Für die vorgestellten Analysen sind dies nichtinformative Aprioriverteilungen gemäß den Standardeinstellungen der verwendeten Analysesoftware GeoBUGS [6]. Die 95 % Kredibilitätsintervalle zu den Punktschätzern werden in Onlinetabelle Z4 berichtet.

Die relativen Abweichungen (RRR für sFFR und RR für sMR und sFR) werden auf Karten wie folgt eingefärbt: RR(R) < 0,5 (dunkelblau), 0,5 ≤ RR(R) < 0,75 (blau), 0,75 ≤ RR(R) < 1 (hellblau), 1 ≤ RR(R) < 1,25 (hellrot), 1,25 ≤ RR(R) < 2 (rot) und 2,0 ≤ RR(R) (dunkelrot).

### Daten

Die betrachteten Regionen werden durch die 96 bayerischen Landkreise und kreisfreien Städte definiert. Die dazugehörenden demografischen Daten liefert der statistische Bericht des Bayerischen Landesamtes für Statistik des Jahres 2019 [7]. Vier Altersgruppen werden pro Geschlecht betrachtet: unter 18-Jährige, 18- bis 29-Jährige, 30- bis 64-Jährige und 65-Jährige und Ältere.

Die Daten zum Infektionsgeschehen stammen vom Bayerischen Landesamt für Gesundheit und Lebensmittelsicherheit (LGL). Das LGL legt seinen Statistiken die Falldefinition des Robert Koch-Instituts zugrunde [8]. Die am 02.04.2021 übermittelten Daten wurden quartalsweise für 4 Zeiträume zwischen 01.04.2020 und 31.03.2021 aufbereitet. Dazu wurde die Anzahl an mit/an COVID-19 Verstorbener und Infizierter pro Altersgruppe und Geschlecht für jede Region zusammengefasst. Die Personen mit fehlender Angabe zum Geschlecht oder Alter sind von dieser Analyse ausgeschlossen (*n* = 160 Personen, 0,03 % aller Daten). Das Quartal Q1/20 wurde wegen sporadisch aufgetretener Infektionen und der Etablierung der Meldesysteme nicht betrachtet. Zwischen der Ansteckung und dem Versterben wurde ein zeitlicher Versatz von 14 Tagen angenommen. Das berücksichtigt ein 3‑Wochen-Intervall zwischen der Ansteckung und dem Versterben. Ein solches Vorgehen verwenden auch andere Autoren [9, 10]. So wurden für das Quartal Q4/20 die Todesfälle zwischen 01.10. und 31.12.2020 und die Infektionen zwischen 17.09. und 17.12.2020 zusammengefasst.

Die Kartenvorlage für Bayern stammt vom Open Data Portal Bayern (Bayerische Vermessungsverwaltung – www.geodaten.bayern.de). Die Analysen verwenden die Software R (Version 3.6.3 [11]) und GeoBUGS (Version 1.2 [6]). Die Landkarten wurden mit QGIS (Version 3.10.10 [12]) erstellt. Die Daten und Codes sind in dem OSF-Repositorium unter https://osf.io/p689n/ zu finden.

## Ergebnisse

Für Q2/20 (Q3/20, Q4/20, Q1/21) liegen in Bayern Meldungen über 45.857 (15.852, 212.367, 182.859) Infektionsfälle und 2272 (73, 5255, 5143) Todesfälle vor. Daraus ergibt sich die quartalsspezifische nFFR als 5,0 % (0,5 %, 2,5 %, 2,8 %).

**Figure 1:**
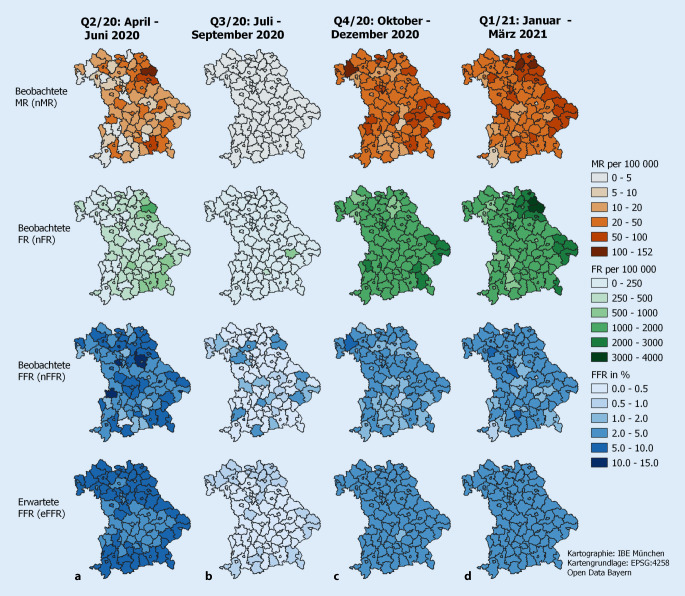

Abb. 1 zeigt das regionale Infektionsgeschehen in Bayern (a: Q2/20, b: Q3/20, c: Q4/20, d: Q1/21). Die dazugehörenden deskriptiven Statistiken (regionales Minimum, Median, Maximum) finden sich im Onlinetabelle Z2. Die erste und zweite Zeile der Abbildung zeigen die naiven infektionsbezogenen Mortalitäts- (nMR) und Fallraten (nFR) pro 100.000 Einwohner. Aus der Division der ersten Zeile durch die zweite Zeile ergeben sich die regionalen naiven Fallfatalitätsraten (nFFR), die in der dritten Zeile dargestellt sind. Die vierte Zeile zeigt aufgrund der 4 unterschiedlichen Standards die jeweils erwarteten FFR (eFFR). Die entsprechenden sFFR-Werte (Division von Zeile 3 durch Zeile 4) zeigt die erste Zeile der Abb. 3.

Die beobachteten Inzidenzen in Zeile 2 der Abb. 1 sind Quartalsinzidenzen über einen Zeitraum von etwa 12 Wochen. Eine Division dieser Werte durch 12 führt zu einem Mittelwert der regionalen 7‑Tages-Inzidenz pro 100.000 Einwohner.

In Q2/20 (Q3/20, Q4/20, Q1/21) wurden regionale nFFR-Werte zwischen 0 % und 12,5 % (0–5,8 %, 0,7–8,3 %, 0,7–5,9 %) beobachtet. In Q3/20 wurden zwar 15.852 Infektionen gemeldet, davon verstarben aber nur 73 Infizierte und es gab insgesamt 60 Regionen (63 % aller 96 Regionen) ohne gemeldete Todesfälle.

Innerhalb eines Quartals reflektieren die Unterschiede der regionalen eFFR-Werte demografische Unterschiede. Sie zeigen jüngere Populationen im Zentrum Bayerns und ältere Populationen in den Randgebieten. Unter der Annahme stabiler demografischer Verhältnisse in Bayern über das betrachtete Jahr spiegeln Quartalsunterschiede Veränderungen im Standard wider. Im Q2/20 (Q3/20, Q4/20, Q1/21) liegen die eFFR-Werte zwischen 4,0 % und 6,1 % (0,4–0,6 %, 1,9–3,2 %, 2,2–3,6 %). Onlinetabelle Z3 gibt eine detaillierte Beschreibung der verwendeten Standards und deren Veränderung über die 4 Quartale.

**Figure 2:**
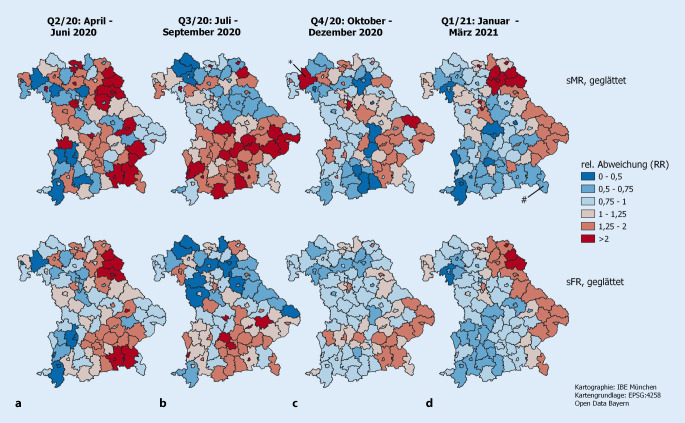

**Figure 3:**
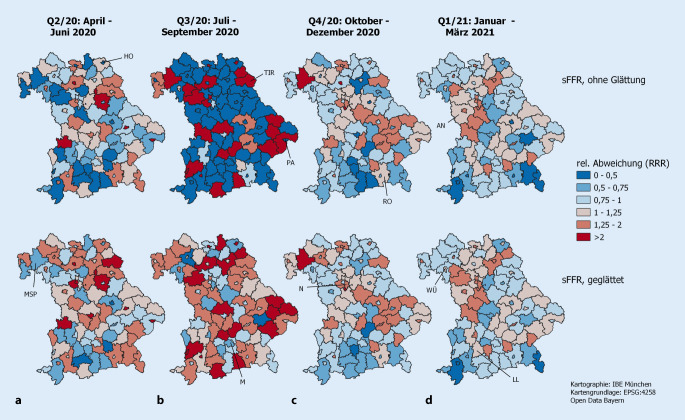

Abb. 2 zeigt die regionalen geglätteten Abweichungen vom bayerischen Standard in Mortalität (sMR, obere Zeile) und dokumentierten Infektionsgeschehen (sFR, untere Zeile) über die 4 Quartale. Beide Maße (sMR und sFR) erlauben eine alternative Interpretation der sFFR als Quotienten der ersten und zweiten Zeile: sFFR_r_ = sMR_r_ / sFR_r_. Ähnliche regionale Färbungen in beiden Karten resultieren in einer sFFR nahe 1, divergente Färbungen erzeugen sFFR-Werte unterhalb oder oberhalb der 1. Daraus entsteht Abb. 3. Abb. 2 zeigt deutliche Divergenzen zwischen der Infektions- und Mortalitätsentwicklung. Abweichungen vom bayerischen Standard der sMR erscheinen optisch ausgeprägter als die der sFR. Detaillierte quantitative Informationen dazu sind in Onlinetabelle Z4 zu finden.

Die unterfränkische Region Bad Kissingen ist in Abb. 2c (obere Karte) mit einem Stern (*) markiert. Die rote Farbe deutet auf eine mehr als zweifache Mortalität im Vergleich zur bayernweiten standardisierten Mortalität. Die dazugehörige sFR (untere Karte in Abb. 2c) zeigt einen geringeren Wert als der Standard. Die erhöhte Mortalität kombiniert mit nicht erhöhtem Infektionsgeschehen führt zu einer sFFR > 1 (Abb. 3c). Im Berchtesgadener Land (Oberbayern, in Abb. 2d mit einer Raute (#) markiert) ist die Sterblichkeit etwas geringer, aber die sFR höher als erwartet. Kombiniert führt das zu einer sFFR < 1 (Abb. 3d).

Die obere Reihe aus Abb. 3 zeigt den Quotienten von nFFR- und eFFR-Werten aus Abb. 1. Die untere Reihe aus Abb. 3 basiert auf einer Glättung durch das bivariate BYM-Modell und entspricht in etwa dem Quotienten der ersten und zweiten Zeile der Abb. 2. Beide Abbildungsreihen reflektieren, wo in Bayern SARS-CoV-2-Infizierte ein zum Standard gesehen höheres (Rotfärbung) oder geringeres (Blaufärbung) Sterberisiko haben. Das in Reihe 2 angewendete Modell berücksichtigt regionale Korrelationen zwischen Infektions- und Sterblichkeitsmaßen und erlaubt präzisere Schätzungen der sFFR. Auffällige Unterschiede zwischen beiden Darstellungen ergeben sich vor allem für Q2/20 und Q3/20. Bei der Darstellung zu Q2/20 ist der *Shrinkage*-Effekt der Glättung zu sehen. Bleiben Blau- und Rotmuster etwa erhalten, so werden in der unteren Reihe für die sFFR-Karte zu Q2/20 die Farbtöne blasser, was die Abschwächung darstellt (Shrinkage zu 1 hin). Detaillierte quantitative Informationen dazu sind in Onlinetabelle Z4 zu finden.

In Q3/20 führen die Glättungseffekte zum Umschlag der Kartenfarbe von überwiegend blau nach rot. Einige blaugefärbte Gebiete werden sogar tiefrot. Hier handelt es sich um numerische Artefakte des Verfahrens. In Q3/20 wurden nur 73 Todesfälle unter COVID-19-Erkrankten beobachtet. Viele Regionen haben eine rohe sMR von 0. Aufgrund dieser bayernweit wenigen beobachteten Todesfälle wird das Glättungsverfahren für solche Regionen eine sMR von 1 schätzen. Da sich 15.852 Infektionen über die bayerische Bevölkerung verteilen, ist ausreichend Information vorhanden und lokale rohe sFR-Werte werden weniger durch die Glättung modifiziert (Onlinetabelle Z4).

An der bayerischen Ostgrenze zeigt Abb. 2 für Q4/20 und Q1/21 mehr Infektions- und Sterbefälle als nach bayerischem Standard erwartet. Beide Zahlen entwickeln sich in die gleiche Richtung und deshalb erscheinen auf den entsprechenden sFFR-Karten in Abb. 3 keine wesentlich von 1 verschiedenen Werte.

Zeigt Abb. 3 für Q2/20 noch ein farblich kontrastreicheres Bild als für Q4/20 und Q1/21, so bedeutet dies, dass sich die Mortalität von Infizierten in Bayern in der zweiten Welle regional angeglichen hat.

Tab. 2 zeigt die FFR-Werte in ausgewählten bayerischen Regionen für die 4 Quartale. Gezeigt werden die Werte für eine Region pro Regierungsbezirk (Landkreis Hof in Oberfranken, Tirschenreuth in Oberpfalz, Passau in Niederbayern, Rosenheim in Oberbayern, Landsberg am Lech in Schwaben, Ansbach in Mittelfranken und Main-Spessart in Unterfranken) und für die 3 bayerischen Städte München, Nürnberg und Würzburg. Zur Orientierung sind diese Regionen in Abb. 3 gekennzeichnet.

**Table 2:** 

Region (Einwohnerzahl)	Quartal	Infektionen (*n*)	Todesfälle (*n*)	nFFR (%)	eFFR (%)	sFFR roh	sFFR geglättet
Hof (94.801)	Q2/20	417	30	7,194	6,051	1,189	1,325
	Q3/20	99	0	0	0,601	0	0,599
	Q4/20	1432	38	2,654	3,153	0,842	0,922
	Q1/21	2287	48	2,099	3,523	0,596	0,697
Tirschenreuth (72.046)	Q2/20	1102	101	9,165	5,472	1,675	2,064
	Q3/20	43	1	2,326	0,526	4,423	1,933
	Q4/20	1006	41	4,076	2,790	1,461	1,396
	Q1/21	2508	66	2,632	3,147	0,836	0,918
Passau (192.656)	Q2/20	565	22	3,893	5,346	0,728	0,959
	Q3/20	185	2	1,081	0,510	2,120	1,838
	Q4/20	4832	141	2,918	2,711	1,077	1,131
	Q1/21	5001	167	3,339	3,062	1,090	1,126
Rosenheim (261.330)	Q2/20	2239	175	7,816	5,207	1,501	1,813
	Q3/20	234	1	0,427	0,490	0,872	1,565
	Q4/20	5180	156	3,012	2,614	1,152	1,175
	Q1/21	3529	78	2,210	2,962	0,746	0,789
Landsberg am Lech (120.302)	Q2/20	353	6	1,700	4,931	0,345	0,657
	Q3/20	91	0	0	0,461	0	1,381
	Q4/20	1624	23	1,416	2,449	0,578	0,648
	Q1/21	924	28	3,030	2,787	1,087	1,005
Ansbach (184.591)	Q2/20	600	28	4,667	4,947	0,943	1,191
	Q3/20	61	0	0	0,462	0	1,976
	Q4/20	2333	53	2,272	2,461	0,923	0,966
	Q1/21	2272	69	3,037	2,800	1,085	1,134
Main-Spessart (126.158)	Q2/20	150	4	2,667	5,559	0,480	0,558
	Q3/20	54	1	1,852	0,540	3,427	1,968
	Q4/20	1836	152	8,279	2,840	2,915	2,832
	Q1/21	1408	35	2,486	3,199	0,777	0,853
Stadt München (1.484.226)	Q2/20	6151	200	3,252	4,242	0,766	0,937
	Q3/20	3723	6	0,161	0,382	0,422	0,577
	Q4/20	28.515	546	1,915	2,080	0,920	0,954
	Q1/21	17.321	342	1,974	2,386	0,828	0,859
Stadt Nürnberg (518.370)	Q2/20	1079	49	4,541	4,803	0,946	1,118
	Q3/20	745	3	0,403	0,437	0,922	0,749
	Q4/20	11.748	397	3,379	2,395	1,411	1,471
	Q1/21	10.916	396	3,628	2,725	1,331	1,389
Stadt Würzburg (127.934)	Q2/20	420	33	7,857	4,611	1,704	1,929
	Q3/20	226	1	0,442	0,398	1,113	1,266
	Q4/20	1296	18	1,389	2,302	0,603	0,712
	Q1/21	1122	31	2,763	2,628	1,052	1,086

*nFFR* naive Fallfatalitätsrate, *eFFR* erwartete FFR, *sFFR* standardisierte FFR, *Q* Quartal

Landkreis Tirschenreuth liegt an der Grenze zu Tschechien. Er wurde im Frühjahr 2020 durch die Verhängung der allerersten Ausgangsbeschränkungen bekannt. In Q2/20 wurden 101 Todesfälle unter 1102 Infizierten gemeldet. Das entspricht einer nFFR von 9,2 %. Erwartet wurde ein Wert von eFFR = 5,4 %. Aus diesen Zahlen ergibt sich eine sFFR von 1,7. Durch die Berücksichtigung der Nachbarkreise wurde die sFFR zu 2,1 korrigiert. Die Region hat direkte Nachbarn mit erhöhten Fall- und Todesraten. In Q3/20 verzeichnete die Region einen Todesfall unter 43 Infizierten, was einer nFFR von 2,3 % entspricht. Erwartet wurde eine eFFR von 0,5 %, sodass sFFR = 4,4 ist. Nach der Glättung wurde sFFR zu 1,9. Die instabile Schätzung basierend auf einem Todesfall wurde durch das Glättungsverfahren stabilisiert. Abb. 2 zeigt, dass die sMR in der Region über alle Zeiträume hinweg und die sFR in Q2/20 und Q1/21 erhöht (> 1) sind. Die sFFR (Abb. 3) ist bis auf Q1/21 erhöht. Anfang 2021 sind sowohl Infektions- als auch Todeszahlen erhöht, wodurch die sFFR keine auffälligen Werte aufzeigt.

Abb. 1**und** 2 demonstrieren die Komponenten, aus denen sich die sFFR ergibt. Vor allem zeigt Abb. 3 regionale Heterogenität des relativen Risikos, als dokumentierter Infektionsfall an/mit COVID-19 zu versterben. Mehr Regionen mit höherem Sterberisiko für Infizierte liegen im Norden Bayerns.

**Figure 4:**
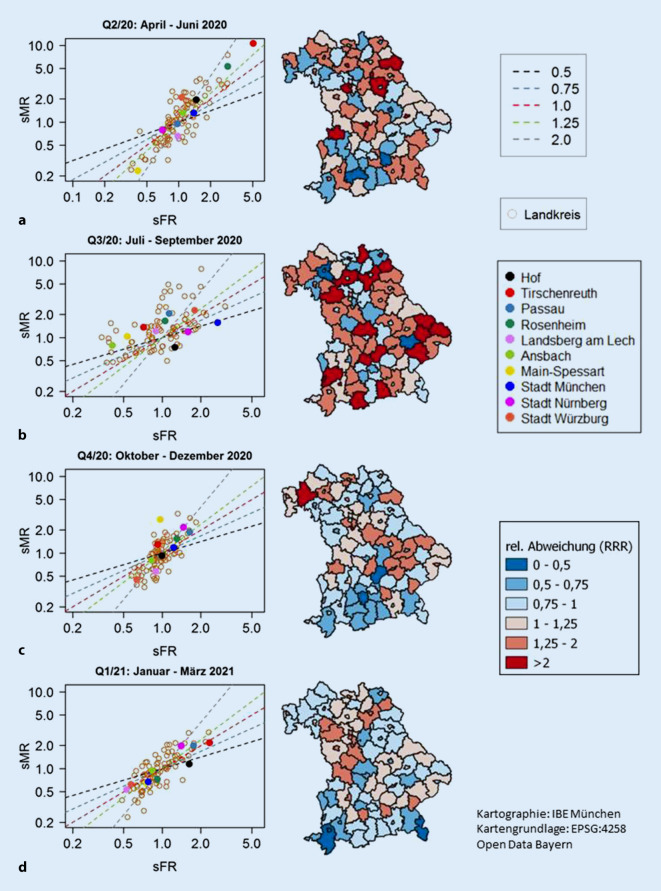

Abb. 4 zeigt in der linken Spalte quartalsspezifische Streudiagramme regionaler sMR- und sFR-Werte. Die dargestellten Geraden entsprechen Verhältnissen zwischen beiden Größen und repräsentieren sFFR-Werte. Die Steigungen dieser Geraden sind in der rechten Spalte in den Karten farblich wiedergegeben. Wo das spezifische (sFR_r_, sMR_r_)-Paar auf dieser Geraden genau liegt, kann auf der Karte nicht erkannt werden. Im Vergleich zu den Werten in Q2/20 und Q3/20 zeigen das regionale Infektionsgeschehen und die regionale Mortalität in Q4/20 und Q1/21 vergleichbare Abweichungen vom jeweiligen Standard: Die Farben in den Karten werden blasser.

## Diskussion

Das Konzept der regionalen standardisierten Fallfatalitätsrate sFFR_r_ ermöglicht den Vergleich der in einer Region beobachteten mit der (aufgrund der regionalen Population und eines gegebenen Standards) erwarteten fallbezogenen Sterblichkeit. Differenzielle Inzidenzen und Mortalitätsraten zwischen verschiedenen Populationsschichten sowie uneinheitliche regionale Verteilungen dieser Populationsgruppen werden bei der Berechnung der sFFR_r_ berücksichtigt. Der Wert der sFFR approximiert das regionale relative Risiko, als Infizierter an/mit COVID-19 zu versterben.

Der gewählte Standard bestimmt die Interpretation der sFFR_r_. Externe Standards (basierend auf dem Infektionsgeschehen anderer Nationen, andere Bundesländer) erlauben Vergleiche mit externen Verhältnissen. Für diese Arbeit wurde ein interner Standard gewählt: populationsstratifizierte Inzidenz- und Sterbezahlen für das Bundesland Bayern aggregiert über seine 96 Regionen. Damit lässt sich Heterogenität/Homogenität innerhalb Bayerns verdeutlichen. In seiner Landesverfassung verspricht Bayern die Förderung und Sicherung „gleichwertiger Lebensverhältnisse und Arbeitsbedingungen in ganz Bayern, in Stadt und Land“ (Artikel 3 (2), Satz 2). Damit ist Homogenität der sFFR_r_-Werte in bayerischen Regionen ein anzustrebendes Ziel.

Formal ist die sFFR_r_ auch das Verhältnis zweier regionaler standardisierter Raten: der regionalen standardisierten infektionsbedingten Mortalitätsrate sMR_r_ und der standardisierten Fallrate sFR_r_. Die sFFR_r_ beschreibt, welche der beiden Aspekte, Infektionsgeschehen oder infektionsspezifische Mortalität, sich weiter vom entsprechenden Standard entfernen. Eine sFFR kleiner als 1 impliziert eine höhere standardisierte Inzidenzrate im Vergleich zur standardisierten Fallsterblichkeit und vice versa.

Hieraus ergeben sich folgende Interpretationsschwierigkeiten: Eine gleichsinnige Zunahme im Infektionsgeschehen und in der Fallsterblichkeit verändert die sFFR_r_ nicht. Dieses Phänomen hat sich in den östlichen Regionen Bayerns in Q4/20 und Q1/21 gezeigt (Abb. 2**und** 3). Trotz unterschiedlicher regionaler (sFR, sMR)-Werte bleibt das infektionsbedingte relative Sterberisiko vergleichbar.

Die sFFR hat folgende Eigenschaften: Ist die Infektionsaktivität zwischen 2 Regionen sehr unterschiedlich, ohne dass sich die regionalen Verhältnisse zwischen infektionsassoziierten Sterbefällen und dokumentierten Infektionen unterscheiden, liegen 2 gleiche sFFR-Werte vor: Beide nFFR- und eFFR-Werte unterscheiden sich nicht. Wird in einer Region mehr getestet (Schulöffnungen, Modellregion) und werden dadurch mehr Infektionen erkannt, die aber leicht verlaufen und damit die Mortalität absolut nicht beeinflussen, so wird in dieser Region die nFFR gegenüber einer Region mit weniger Testung reduziert, die eFFR bleibt konstant und somit reduziert sich die sFFR. Anders betrachtet: Die sMR bleibt in diesem Szenario zwischen den Regionen gleich, während sich die sFR erhöht und somit die sFFR reduziert. Führt eine hohe Infektionsaktivität zu einer Überforderung der intensivmedizinischen Versorgung und zu erhöhten Sterbezahlen, so nimmt die sFFR in dieser Region zu, denn die nFFR wird gegenüber Vergleichsregionen ohne Versorgungsprobleme bei gleichbleibender eFFR wachsen. Bleibt das Infektionsgeschehen konstant, wobei eine aggressive Mutation mit schwereren Krankheitsverläufen in einer Region die vorherrschende Virusvariante verdrängt, führt dies zu einer Zunahme der nFFR bei gleichbleibender eFFR oder wachsender sMR bei konstanter sFR.

Die statistische Analyse dieser Daten wird mit hierarchischen bayesianischen Modellen durchgeführt. Pritzkuleit et al. [13] sehen darin Standardwerkzeuge der geografischen Epidemiologie zur Glättung von Karten. Die Methoden erlauben die Einbeziehung von Regressionskomponenten und damit die Möglichkeit, für regionale Faktoren (Deprivationsindex, regionale Mobilität, Impfquote) zu adjustieren. Für die simultane Analyse von Infektionsinzidenzen und Mortalität wird ein bivariates Besag-York-Mollié-Modell verwendet [2, 5]. Solche Modelle können mit Standardsoftware (GeoBUGS) berechnet werden.

In dieser Arbeit werden die Ergebnisse aus 4 Quartalen berichtet. Karten mit variabler Karteneinfärbung bietet eine Website^1^ der Universität München. Eine variable Zeitskala ist aufgrund des Rechenaufwandes für die Kartenerstellung nicht einfach umzusetzen.

Es gibt viele kartenbasierte Monitoringsysteme für die COVID-19-Pandemie, z. B. [14–18], aber nur wenige erlauben die simultane Betrachtung von regionalen und zeitlichen Veränderungen. In der Regel zeigen die Karten absolute Maße zu Infektionsfällen und Mortalität. Eine Darstellung von standardisierten Fall- oder Mortalitätsraten oder eine kombinierte standardisierte Betrachtung über Raum und Zeit scheint nicht zu existieren. Interessant wäre ein Dashboard, bei dem für ein interessierendes Land Vergleiche mit anderen Ländern über eine Standardisierung der Infektions- und infektionsbezogenen Todesfälle durchgeführt werden könnten.

Das Prinzip der sFFR lässt sich auch auf andere Krankheiten anwenden: kardiovaskuläre Morbidität und Mortalität, Tumorinzidenz und tumorbedingte Sterblichkeit. In solchen Krankheitsfeldern ist Disease Mapping bereits Standard [13, 19–23].

Stärken dieser Arbeit sind die gute Qualität der Daten des LGL Bayern und die Verwendung von hierarchischen Bayes-Modellen zu deren Verarbeitung.

Limitation dieses Beitrags ist einerseits die Verwendung von Daten, die sich allein auf testbasierte Falldefinitionen beziehen und die regionalen Dunkelziffern der Infizierten nicht berücksichtigen. Entsprechende Korrekturen sind theoretisch möglich, führen jedoch zu noch komplexeren Modellen. Weiterhin findet keine Korrektur der Daten hinsichtlich der Testqualität statt, was auch in einem komplexeren Modell möglich wäre. Drittens sind die Möglichkeiten der Darstellung der geografischen Information über die Zeit eingeschränkt. Für die Implementierung eines entsprechenden Dashboards ist die Arbeit einer Berliner Gruppe zur Dynamisierung komplexer raumzeitlicher Phänomene ein wichtiges Vorbild [24]. Der Gruppe gelingt durch Einsatz schneller Algorithmen eine in realer Zeit mögliche Berechnung komplexer Karten. Diese Algorithmen können jedoch bivariate Endpunkte, so wie in dieser Arbeit verwendet (sFR, sMR), noch nicht bearbeiten.

Bisher war das Infektionsgeschehen im Zentrum der öffentlichen Wahrnehmung. Mit den sFFR-Karten wird zu diesem Geschehen die Entwicklung von regionalen infektionsbezogenen Sterbezahlen gekoppelt. Wichtig ist die Detektion von Regionen mit einer starken Entwicklung der Sterbe- im Vergleich zu den Infektionszahlen. Damit werden Regionen identifiziert, die zusätzlich zum Management der Infektionsausbreitung Maßnahmen zur Kontrolle der Sterblichkeit benötigen.

## Fazit

Eine über Bayern und für alle Populationen gemeinsame konstante FFR führt zu einer regionalen sFFR von 1. Gelten bayernweite konstante FFRs für spezifische Populationsstrata, sind die Strata aber regional unterschiedlich verteilt, so spiegelt die Heterogenität der sFFR Populationsheterogenität wider. Diese sollte aber über die betrachtete Zeit hinweg homogen sein. Die über die Zeit betrachtete Heterogenität in der sFFR wird dem Infektionsgeschehen zugeschrieben. Heterogenität ergibt sich aus regionalen Einflüssen auf die Infektionshäufigkeiten (mehr Testen, Hotspots, Schulöffnungen, Kontaktbeschränkungen, Impfungen) oder die Versorgung der Erkrankten (Erreichen von Behandlungslimits, nicht vorhandene Versorgungsmöglichkeiten, Einführen von effektiven Therapien, schwächere Krankheitsverläufe durch Impfung). Solche Phänomene können mit der sFFR dargestellt werden. Es wird hier nicht versucht, diese zu erklären.

## Supplementary Information





## Footnotes

1 https://covid19.ibe.med.uni-muenchen.de/public/.
