# Supplementary material for: Regional monitoring of infections by means of standardized case fatality rates using the example of SARS-CoV-2 in Bavaria
Source: Bundesgesundheitsblatt Gesundheitsforschung Gesundheitsschutz. 2021 Aug 12;64(9):1146–56. [Article in German] doi: 10.1007/s00103-021-03397-8 (PMC8358915; doi:10.1007/s00103-021-03397-8)
Supplement: Supplementary file 1 [file 103_2021_3397_MOESM1_ESM.pdf]

Bundesgesundheitsblatt 9/2021

Onlinematerial zum Beitrag:

Regionales Monitoring von Infektionen mittels standardisierter Fallfatalitätsraten am Beispiel von SARS-CoV-2 in Bayern

Kirsi Manz[1], Ulrich Mansmann[1,2]

[1] Institut für Medizinische Informationsverarbeitung, Biometrie und Epidemiologie (IBE), Ludwig-Maximilians-Universität München, München, Bayern, Deutschland
[2] Pettenkofer School of Public Health (PSPH), Ludwig-Maximilians-Universität München, München, Bayern, Deutschland

Korrespondenzadresse:
Dipl.-Phys. Kirsi Manz, M.Sc.
Institut für Medizinische Informationsverarbeitung, Biometrie und Epidemiologie (IBE)
Ludwig-Maximilians-Universität München
Marchioninistr. 15
81377 München
Deutschland
manz@ibe.med.uni-muenchen.de

Inhalt:

Tabelle Z1: Beispiele zur standardisierten Fallfatalitätsrate (sFFR).

Tabelle Z2: Deskriptive Statistiken zu den regionalen Daten für die vier beobachteten Quartale.

Tabelle Z3: Für die Standardisierung verwendete bayerische Standards für die vier beobachteten Quartale.

Tabelle Z4: Regionale Daten für die vier beobachteten Quartale.

Tabelle Z1: Beispiele zur standardisierten Fallfatalitätsrate (sFFR).

**Standard**			
Gruppe	Infektionen	Infektionsbedingte Todesfälle	Pro XXX Einwohner
A1	50	1	1000
A2	100	10	1000
A3	150	75	1000

**Kreis 1**			
Gruppe	Infektionen	Infektionsbedingte Todesfälle	Einwohneranzahl
A1	1000	20	20000
A2	5000	400	20000
A3	4000	2500	10000

sFR.1	2,22222222	Standardisierte Infektions- und Mortalitätsraten zeigen eine starke Abweichung vom Standard nach oben.
sMR.1	3,01030928	Infektions- und Sterbefälle sind größer als erwartet.
sFFR.1	1,35463918	Abweichung der Todesfälle vom Standard ist größer als Abweichungen der Infektionszahlen vom Standard.

**Kreis 2**			
Gruppe	Infektionen	Infektionsbedingte Todesfälle	Einwohneranzahl
A1	1000	20	10000
A2	5000	400	10000
A3	4000	2500	10000

Kreis 1 und *Kreis 2* zeigen absolut das gleiche Infektionsgeschehen und infektionsbasierte Mortalität.

sFR.2	3,33333333	Standardisierte Infektions- und Mortalitätsraten zeigen eine starke Abweichung vom Standard nach oben.
sMR.2	3,39534884	Infektions- und Sterbefälle sind größer als erwartet.
sFFR.2	1,01860465	Abweichung der Todesfälle vom Standard ist vergleichbar der Abweichungen der Infektionszahlen vom Standard.

**Kreis 3**			
Gruppe	Infektionen	Infektionsbedingte Todesfälle	Einwohneranzahl
A1	1000	20	10000
A2	5000	100	10000
A3	4000	80	10000

Kreis 3 hat Infektionsgeschehen wie *Kreis 2* aber eine viel geringere infektionsbedingte Mortalität (nur 2%) in jeder Gruppe.

sFR.3	3,33333333	Standardisierte Infektionsrate zeigt eine starke Abweichung nach oben.
sMR.3	0,23255814	Standardisierte Mortalitätsrate ist aufgrund der geringeren Mortalität (nur 2%) kleiner als 1.
sFFR.3	0,06976744	Abweichung der Todesfälle vom Standard ist viel geringer als die Abweichungen der Infektionszahlen vom Standard.

**Kreis 4**			
Gruppe	Infektionen	Infektionsbedingte Todesfälle	Einwohneranzahl
A1	500	10	10000
A2	1500	150	20000
A3	3000	1500	30000

Kreis 4 hat trotz älterer Bevölkerung ein geringes Infektionsgeschehen aber eine infektionsbedingte Mortalität wie der *Standard* (2%, 10%, 50%).

sFR.4	0,71428571	Standardisierte Infektionsrate zeigt eine starke Abweichung nach unten.
sMR.4	0,67479675	Standardisierte Mortalitätsrate zeigt eine starke Abweichung nach unten.
sFFR.4	0,94471545	Abweichung der Todesfälle vom Standard ist vergleichbar zu Abweichungen der Infektionszahlen vom Standard.

**Kreis 5**			
Gruppe	Infektionen	Infektionsbedingte Todesfälle	Einwohneranzahl
A1	500	10	10000
A2	1500	100	20000
A3	3000	1000	30000

Kreis 5 hat trotz älterer Bevölkerung ein geringes Infektionsgeschehen und eine geringere infektionsbedingte Mortalität wie der *Standard*.

sFR.5	0,71428571	Standardisierte Infektionsrate zeigt eine starke Abweichung nach unten.
sMR.5	0,45121951	Standardisierte Mortalitätsrate is aufgrund der geringeren Mortalität kleiner als 1.
sFFR.5	0,63170732	Abweichung der Todesfälle vom Standard ist viel geringer als die Abweichungen der Infektionszahlen vom Standard.

Tabelle Z2: Deskriptive Statistiken zu den regionalen Daten über die beobachteten Quartale.

Die Daten wurden kumulativ für die 3 Monate pro Analysequartal aufbereitet.

	**Q2/20: April – Juni 2020**			**Q3/20: Juli – September 2020**			**Q4/20: Oktober – Dezember 2020**			**Q1/21: Januar – März 2021**		
	Min	Median	Max	Min	Median	Max	Min	Median	Max	Min	Median	Max
nMR (per 100.000)	0	13,4	140,2	0	0	9,0	9,4	35,3	139,1	7,2	36,3	151,4
nFR (per 100.000)	103,8	304,0	1529,6	22,2	86,5	688,8	868,1	1457,9	2829,1	712,3	1292,8	3481,1
nFFR (%)	0	4,6	12,5	0	0	5,8	0,7	2,3	8,3	0,7	2,7	5,9
eFFR (%)	4,0	5,1	6,1	0,4	0,5	0,6	1,9	2,6	3,2	2,2	2,9	3,6
sMR	0	0,8	7,3	0	0	15,0	0,2	0,9	3,3	0,2	0,9	3,1
sFR	0,3	0,9	4,3	0,2	0,7	5,7	0,5	0,9	1,8	0,5	0,9	2,5
sFFR	0	0,9	2,7	0	0	11,4	0,3	0,9	2,9	0,2	0,9	2,0

nMR=naive Mortalitätsrate, nFR=naive Fallrate, nFFR=naive Fallfatalitätsrate, eFFR=erwartete FFR, sMR=standardisierte Mortalitätsrate, sFR=standardisierte Fallrate, sFFR=standardisierte FFR.

Tabelle Z3: Für die Standardisierung verwendete bayerische Standards für die vier beobachteten Quartale.

Standard			Q2/20: April - Juni 2020		Q3/20: Juli - September 2020		Q4/20: Oktober - Dezember 2020		Q1/21: Januar - März 2021	
**Gruppe**	**Geschlecht**	**Alters-gruppe**	**Infektionen per 1.000 Einwohner**	**Infektionsbedingte Todesfälle per 1.000 Einwohner**	**Infektionen per 1.000 Einwohner**	**Infektionsbedingte Todesfälle per 1.000 Einwohner**	**Infektionen per 1.000 Einwohner**	**Infektionsbedingte Todesfälle per 1.000 Einwohner**	**Infektionen per 1.000 Einwohner**	**Infektionsbedingte Todesfälle per 1.000 Einwohner**
M1	männlich	unter 18	1,1007	0,0009	1,1910	0,0009	12,4338	0,0009	9,2137	0,0009
M2	männlich	18 - 29	3,8656	0,0020	2,9480	0,0010	22,1241	0,0010	16,9879	0,0010
M3	männlich	30 - 64	3,6220	0,0370	1,1710	0,0025	16,9490	0,0537	14,4712	0,0758
M4	männlich	65 und älter	4,1822	0,8866	0,3498	0,0302	12,3955	2,0139	12,4173	1,9669
W1	weiblich	unter 18	1,1586	0,0009	1,1681	0,0009	12,1908	0,0009	9,2677	0,0009
W2	weiblich	18 - 29	4,3461	0,0011	2,5226	0,0011	22,6174	0,0011	18,3580	0,0011
W3	weiblich	30 - 64	4,0481	0,0177	1,1021	0,0003	16,9480	0,0256	14,9478	0,0294
W4	weiblich	65 und älter	4,1546	0,6887	0,2981	0,0160	13,7195	1,7273	13,9331	1,6348

Tabelle Z4: Regionale Daten für die vier beobachteten Quartale.

Z4.1. Daten für April bis Juni 2020 (Q2/20)

Landkreis	Population	Tode beob.	Tode erw.	sMR roh	sMR glatt	Inf beob.	Inf erw.	sFR roh	sFR glatt	nFFR	eFFR	sFFR roh	sFFR glatt	95% KI untere	95% KI obere
LK Aichach-Friedberg	134655	18	22,8	0,79	1,074	375	464,3	0,81	0,9594	4,8	4,92	0,98	1,111	0,7045	1,652
LK Altoetting	111516	49	20,5	2,39	3,244	590	387,9	1,52	1,807	8,31	5,28	1,57	1,781	1,22	2,483
LK Amberg-Sulzbach	103049	51	18,6	2,74	3,633	482	361,3	1,33	1,592	10,58	5,15	2,06	2,266	1,577	3,136
LK Ansbach	184591	28	31,7	0,88	1,322	600	640,9	0,94	1,1	4,67	4,95	0,94	1,191	0,7701	1,712
LK Aschaffenburg	174200	30	32,1	0,94	1,336	527	609,4	0,86	1,018	5,69	5,26	1,08	1,303	0,8581	1,862
LK Augsburg	253468	10	44,3	0,23	0,3249	353	876,6	0,4	0,4805	2,83	5,05	0,56	0,6695	0,3841	1,054
LK Bad Kissingen	103235	17	21,3	0,8	1,076	249	364,6	0,68	0,8135	6,83	5,84	1,17	1,314	0,8097	2,032
LK Bad Toelz-Wolfratshausen	127917	8	23,1	0,35	0,6395	412	444,4	0,93	1,087	1,94	5,2	0,37	0,583	0,3352	0,9216
LK Bamberg	147163	29	24,3	1,19	1,511	409	510,6	0,8	0,9606	7,09	4,76	1,49	1,561	1,039	2,242
LK Bayreuth	103664	26	19,7	1,32	1,77	417	363,8	1,15	1,363	6,24	5,42	1,15	1,288	0,8312	1,862
LK Berchtesgadener Land	105929	21	20,9	1	1,31	296	373,7	0,79	0,9497	7,09	5,6	1,27	1,369	0,8254	2,122
LK Cham	127998	18	23,4	0,77	1,077	395	449,5	0,88	1,04	4,56	5,2	0,88	1,027	0,6281	1,553
LK Coburg	86747	29	17	1,71	2,355	352	305,4	1,15	1,361	8,24	5,55	1,48	1,717	1,144	2,463
LK Dachau	154899	25	24,6	1,01	1,764	901	534,6	1,69	1,96	2,77	4,61	0,6	0,8916	0,5709	1,276
LK Deggendorf	119478	11	20,9	0,53	0,7819	309	419,5	0,74	0,873	3,56	4,97	0,72	0,8863	0,5083	1,4
LK Dillingen a.d.Donau	96562	28	16,6	1,68	2,126	263	335,1	0,78	0,9444	10,65	4,97	2,14	2,237	1,4	3,349
LK Dingolfing-Landau	96683	9	15,9	0,57	0,6477	205	336,5	0,61	0,7425	4,39	4,72	0,93	0,8645	0,4678	1,443
LK Donau-Ries	133783	17	22,9	0,74	1,053	343	464,1	0,74	0,8733	4,96	4,93	1,01	1,195	0,747	1,789
LK Ebersberg	143649	5	22,9	0,22	0,5503	483	492,3	0,98	1,146	1,04	4,64	0,22	0,4751	0,2471	0,7805
LK Eichstaett	132881	16	20,3	0,79	0,9794	300	454,4	0,66	0,7948	5,33	4,46	1,2	1,222	0,7527	1,865
LK Erding	138182	9	20,4	0,44	0,9026	594	475,4	1,25	1,457	1,52	4,3	0,35	0,6129	0,3458	0,9805
LK Erlangen-Hoechstadt	137262	10	24,1	0,42	0,541	237	474	0,5	0,6013	4,22	5,07	0,83	0,8906	0,5088	1,424
LK Forchheim	116203	2	20,3	0,1	0,3182	198	404,6	0,49	0,5737	1,01	5,02	0,2	0,5495	0,2721	0,938
LK Freising	180007	30	25	1,2	1,795	851	621,3	1,37	1,61	3,53	4,02	0,88	1,104	0,7228	1,591
LK Freyung-Grafenau	78362	10	14,7	0,68	0,9147	188	276,8	0,68	0,8098	5,32	5,3	1	1,123	0,6297	1,83

LK Fuerstenfeldbruck	219311	32	39,8	0,8	1,202	879	759,7	1,16	1,362	3,64	5,24	0,7	0,8743	0,5825	1,239
LK Fuerth	117853	55	22,7	2,42	3,392	514	412,2	1,25	1,472	10,7	5,51	1,94	2,288	1,612	3,158
LK Garmisch-Partenkirchen	88424	17	18,4	0,92	1,223	285	313,3	0,91	1,084	5,96	5,88	1,02	1,123	0,6854	1,71
LK Guenzburg	127027	3	21,1	0,14	0,4354	234	440,6	0,53	0,613	1,28	4,78	0,27	0,7021	0,3499	1,189
LK Hassberge	84384	6	15,2	0,4	0,5344	152	294,8	0,52	0,6213	3,95	5,15	0,77	0,853	0,4505	1,429
LK Hof	94801	30	20,4	1,47	1,962	417	337,3	1,24	1,471	7,19	6,05	1,19	1,325	0,8827	1,898
LK Kelheim	123058	28	19,7	1,42	1,894	441	424,8	1,04	1,235	6,35	4,64	1,37	1,521	0,9879	2,198
LK Kitzingen	91155	4	16,2	0,25	0,494	185	318,5	0,58	0,6832	2,16	5,08	0,43	0,7155	0,3645	1,215
LK Kronach	66743	3	13,6	0,22	0,5026	162	238,1	0,68	0,7991	1,85	5,73	0,32	0,6226	0,3134	1,08
LK Kulmbach	71566	9	14,5	0,62	0,9553	234	254,3	0,92	1,082	3,85	5,7	0,67	0,8768	0,4959	1,399
LK Landsberg a.Lech	120302	6	20,5	0,29	0,6529	353	416	0,85	0,9852	1,7	4,93	0,34	0,6568	0,3735	1,03
LK Landshut	159895	18	25,8	0,7	1,154	650	551,4	1,18	1,379	2,77	4,68	0,59	0,8288	0,5251	1,216
LK Lichtenfels	66776	16	12,8	1,25	1,843	291	235,7	1,23	1,442	5,5	5,44	1,01	1,269	0,776	1,893
LK Lindau	81981	6	15,6	0,38	0,7537	220	286,9	0,77	0,8882	2,73	5,43	0,5	0,8438	0,427	1,458
LK Main-Spessart	126158	4	24,7	0,16	0,2331	150	444,6	0,34	0,4133	2,67	5,56	0,48	0,5583	0,2816	0,9798
LK Miesbach	100010	11	18,9	0,58	1,142	554	349,6	1,58	1,842	1,99	5,41	0,37	0,614	0,3592	0,9535
LK Miltenberg	128743	5	23,5	0,21	0,5084	293	450	0,65	0,7549	1,71	5,22	0,33	0,6678	0,3405	1,115
LK Muehldorf a.Inn	115872	22	19,7	1,12	1,515	494	401,9	1,23	1,459	4,45	4,9	0,91	1,03	0,656	1,511
LK Muenchen	350473	82	60,5	1,36	1,791	1330	1207,6	1,1	1,315	6,17	5,01	1,23	1,351	0,9822	1,799
LK Neu-Ulm	175204	25	30,1	0,83	1,165	413	608,8	0,68	0,8019	6,05	4,94	1,23	1,441	0,9001	2,151
LK Neuburg-Schrobenhausen	97303	17	16	1,07	1,574	330	336	0,98	1,15	5,15	4,75	1,08	1,357	0,8386	2,038
LK Neumarkt i.d.OPf.	134573	17	22,1	0,77	1,049	365	467,2	0,78	0,9282	4,66	4,73	0,98	1,121	0,7034	1,671
LK Neustadt a.d.Aisch-Bad Windsheim	101014	6	18	0,33	0,6477	237	351,8	0,67	0,7857	2,53	5,11	0,5	0,8162	0,4547	1,299
LK Neustadt a.d.Waldnaab	94450	61	17	3,59	5,229	829	330,7	2,51	2,94	7,36	5,13	1,43	1,762	1,258	2,407
LK Nuernberger Land	170792	37	32,2	1,15	1,695	627	596,9	1,05	1,233	5,9	5,39	1,09	1,363	0,9282	1,908
LK Oberallgaeu	156008	4	29,8	0,13	0,2387	162	546,3	0,3	0,3571	2,47	5,46	0,45	0,6621	0,3325	1,143
LK Ostallgaeu	141182	34	25,4	1,34	1,869	487	489,1	1	1,175	6,98	5,19	1,35	1,58	1,063	2,204
LK Passau	192656	22	36,2	0,61	0,9467	565	677,1	0,83	0,9778	3,89	5,35	0,73	0,9587	0,603	1,435
LK Pfaffenhofen a.d.Ilm	128227	20	20,2	0,99	1,261	360	442,2	0,81	0,9744	5,56	4,56	1,22	1,285	0,8083	1,922
LK Regen	77410	11	14,7	0,75	0,8819	177	273	0,65	0,79	6,21	5,38	1,15	1,108	0,6103	1,803

LK Regensburg	194070	14	32,5	0,43	0,5967	465	672,2	0,69	0,823	3,01	4,83	0,62	0,7193	0,4432	1,09
LK Rhoen-Grabfeld	79635	8	15,1	0,53	0,825	192	278,6	0,69	0,8109	4,17	5,42	0,77	1,01	0,5628	1,646
LK Rosenheim	261330	175	47,3	3,7	5,312	2239	908,9	2,46	2,906	7,82	5,21	1,5	1,813	1,389	2,305
LK Roth	126749	6	22,6	0,27	0,5358	271	441,6	0,61	0,717	2,21	5,11	0,43	0,7382	0,4035	1,193
LK Rottal-Inn	121502	40	22	1,82	2,72	701	425,3	1,65	1,927	5,71	5,18	1,1	1,399	0,968	1,943
LK Schwandorf	147872	17	25,9	0,66	1,023	492	517,6	0,95	1,117	3,46	5	0,69	0,9091	0,5686	1,346
LK Schweinfurt	115445	25	21,4	1,17	1,926	497	403,6	1,23	1,423	5,03	5,31	0,95	1,342	0,8679	1,908
LK Starnberg	136667	11	26,2	0,42	0,7507	484	473,2	1,02	1,197	2,27	5,55	0,41	0,6221	0,3759	0,9457
LK Straubing-Bogen	101120	23	17	1,35	2,2	538	350,8	1,53	1,778	4,28	4,85	0,88	1,227	0,8005	1,774
LK Tirschenreuth	72046	101	13,9	7,25	10,57	1102	254,4	4,33	5,078	9,17	5,47	1,67	2,064	1,528	2,728
LK Traunstein	177319	81	33,9	2,39	3,516	1277	621,2	2,06	2,415	6,34	5,46	1,16	1,443	1,05	1,938
LK Unterallgaeu	145341	12	25,9	0,46	0,6343	250	504,6	0,5	0,5902	4,8	5,13	0,94	1,068	0,6438	1,645
LK Weilheim-Schongau	135478	1	24,9	0,04	0,3202	323	470,5	0,69	0,7961	0,31	5,29	0,06	0,3973	0,1961	0,6979
LK Weissenburg-Gunzenhausen	94734	24	17,5	1,37	1,922	331	330,2	1	1,183	7,25	5,29	1,37	1,614	1,044	2,354
LK Wuerzburg	162302	10	29,7	0,34	0,6032	380	566,1	0,67	0,7842	2,63	5,24	0,5	0,7622	0,4451	1,192
LK Wunsiedel i.Fichtelgebirge	72655	33	15,9	2,08	3,199	639	258,5	2,47	2,885	5,16	6,13	0,84	1,1	0,7271	1,567
SK Amberg	42207	3	8,3	0,36	0,6127	85	149,4	0,57	0,6771	3,53	5,52	0,64	0,9003	0,379	1,759
SK Ansbach	41798	8	7,5	1,07	1,518	149	146,2	1,02	1,198	5,37	5,11	1,05	1,26	0,6458	2,165
SK Aschaffenburg	71002	2	12,4	0,16	0,3228	101	249,5	0,4	0,4881	1,98	4,99	0,4	0,658	0,2783	1,284
SK Augsburg	296582	12	48,4	0,25	0,3525	397	1046,3	0,38	0,4519	3,02	4,63	0,65	0,7741	0,4367	1,227
SK Bamberg	77373	25	12,6	1,99	2,511	200	275,3	0,73	0,8754	12,5	4,56	2,74	2,851	1,732	4,448
SK Bayreuth	74783	10	13,3	0,75	1,011	206	270,4	0,76	0,9073	4,85	4,93	0,99	1,108	0,5792	1,87
SK Coburg	41072	2	8	0,25	0,6178	100	146,7	0,68	0,7963	2	5,48	0,36	0,7739	0,3266	1,499
SK Erlangen	112528	9	16,9	0,53	0,8054	242	396	0,61	0,7205	3,72	4,25	0,87	1,108	0,6255	1,812
SK Fuerth	128497	36	20,1	1,79	2,441	428	449,2	0,95	1,129	8,41	4,47	1,88	2,144	1,415	3,095
SK Hof	45825	2	9,1	0,22	0,6069	140	161,6	0,87	1,01	1,43	5,64	0,25	0,6007	0,2543	1,159
SK Ingolstadt	137392	39	21,6	1,81	2,42	470	477,4	0,98	1,17	8,3	4,52	1,83	2,053	1,381	2,92
SK Kaufbeuren	44398	5	8,4	0,59	0,8725	107	155,2	0,69	0,8168	4,67	5,43	0,86	1,065	0,4936	1,906
SK Kempten	69151	8	12,7	0,63	0,7613	86	244	0,35	0,4306	9,3	5,22	1,78	1,762	0,8375	3,153
SK Landshut	73411	7	12,6	0,56	0,8953	272	258,4	1,05	1,238	2,57	4,87	0,53	0,7219	0,3658	1,253

SK Memmingen	44100	0	8	0	0,2893	48	154	0,31	0,369	0	5,2	0	0,7827	0,2845	1,694
SK Muenchen	1484226	200	221	0,9	1,319	6151	5209,8	1,18	1,393	3,25	4,24	0,77	0,9372	0,7089	1,21
SK Nuernberg	518370	49	87,4	0,56	0,7924	1079	1820,2	0,59	0,7022	4,54	4,8	0,95	1,118	0,7672	1,532
SK Passau	52803	15	9,4	1,6	1,864	130	190,5	0,68	0,8336	11,54	4,92	2,35	2,23	1,2	3,723
SK Regensburg	153094	7	22,4	0,31	0,6749	638	544,4	1,17	1,372	1,1	4,11	0,27	0,489	0,2597	0,8041
SK Rosenheim	63551	21	10,7	1,96	2,803	511	222,3	2,3	2,704	4,11	4,82	0,85	1,028	0,6246	1,586
SK Schwabach	40981	2	7,5	0,27	0,5344	74	142,6	0,52	0,6136	2,7	5,24	0,52	0,8663	0,3982	1,584
SK Schweinfurt	53426	12	10,5	1,14	1,506	175	187,8	0,93	1,107	6,86	5,59	1,23	1,351	0,7033	2,235
SK Straubing	47791	47	8,6	5,45	7,43	418	169,3	2,47	2,915	11,24	5,09	2,21	2,53	1,733	3,59
SK Weiden i.d.OPf.	42743	18	8,2	2,19	3,099	330	151,5	2,18	2,558	5,45	5,43	1,01	1,204	0,7251	1,877
SK Wuerzburg	127934	33	21,4	1,54	2,089	420	464,2	0,9	1,074	7,86	4,61	1,7	1,929	1,223	2,872

sMR=standardisierte Mortalitätsrate, Inf = Infektionen, sFR= standardisierte Fallrate, nFFR=naive Fallfatalitätsrate, eFFR=erwartete FFR, KI = Kredibilitätsintervall.

Z4.2. Daten für Juli bis September 2020 (Q3/20)

**Landkreis**	**Population**	**Tode beob.**	**Tode erw.**	**sMR roh**	**sMR glatt**	**Inf beob.**	**Inf erw.**	**sFR roh**	**sFR glatt**	**nFFR**	**eFFR**	**sFFR roh**	**sFFR glatt**	**95% KI untere**	**95% KI obere**
LK Aichach-Friedberg	134655	0	0,7	0	1,358	86	160,3	0,54	0,7697	0	0,46	0	1,793	0,3431	4,992
LK Altoetting	111516	0	0,7	0	1,214	145	131,3	1,1	1,511	0	0,5	0	0,8112	0,1253	2,64
LK Amberg-Sulzbach	103049	0	0,6	0	0,6457	32	121,8	0,26	0,3998	0	0,49	0	1,67	0,2433	5,403
LK Ansbach	184591	0	1	0	0,7927	61	221,4	0,28	0,4077	0	0,46	0	1,976	0,3422	5,909
LK Aschaffenburg	174200	1	1	0,98	1,171	116	203,1	0,57	0,7906	0,86	0,5	1,71	1,492	0,2625	4,493
LK Augsburg	253468	1	1,4	0,7	1,365	265	299,6	0,88	1,219	0,38	0,47	0,79	1,13	0,3024	2,923
LK Bad Kissingen	103235	0	0,7	0	0,4705	29	117,1	0,25	0,3709	0	0,57	0	1,306	0,1392	4,617
LK Bad Toelz-Wolfratshausen	127917	0	0,7	0	1,546	197	150,8	1,31	1,791	0	0,49	0	0,8724	0,1311	2,5
LK Bamberg	147163	2	0,8	2,53	1,538	47	175,9	0,27	0,3985	4,26	0,45	9,48	3,964	0,7973	13,5
LK Bayreuth	103664	0	0,6	0	1,007	23	119,3	0,19	0,3368	0	0,52	0	3,11	0,4282	10,17
LK Berchtesgadener Land	105929	0	0,7	0	1,026	117	124,2	0,94	1,288	0	0,53	0	0,8022	0,09215	2,681

LK Cham	127998	0	0,7	0	0,7149	68	151,7	0,45	0,6319	0	0,49	0	1,149	0,2008	3,326
LK Coburg	86747	0	0,5	0	0,7721	74	99,1	0,75	1,011	0	0,54	0	0,7765	0,1152	2,324
LK Dachau	154899	7	0,8	8,72	7,84	277	187,8	1,47	2,016	2,53	0,43	5,91	3,914	0,9701	10,58
LK Deggendorf	119478	2	0,7	2,99	2,486	112	144,8	0,77	1,07	1,79	0,46	3,86	2,362	0,5565	7,091
LK Dillingen a.d.Donau	96562	0	0,5	0	1,104	105	115,9	0,91	1,244	0	0,46	0	0,8969	0,1208	2,842
LK Dingolfing-Landau	96683	0	0,5	0	1,77	666	117,7	5,66	7,702	0	0,44	0	0,2316	0,02186	0,8381
LK Donau-Ries	133783	1	0,7	1,35	1,41	95	160,9	0,59	0,8253	1,05	0,46	2,29	1,736	0,3765	4,999
LK Ebersberg	143649	1	0,7	1,34	2,004	195	173,4	1,12	1,548	0,51	0,43	1,2	1,309	0,2679	3,784
LK Eichstaett	132881	2	0,7	2,99	2,19	110	163,4	0,67	0,9334	1,82	0,41	4,44	2,389	0,5131	7,745
LK Erding	138182	0	0,7	0	1,485	205	170,2	1,2	1,656	0	0,4	0	0,9053	0,1679	2,659
LK Erlangen-Hoechstadt	137262	0	0,8	0	0,7615	118	160,4	0,74	1,003	0	0,48	0	0,7693	0,1329	2,236
LK Forchheim	116203	0	0,7	0	0,6444	81	137,3	0,59	0,8087	0	0,48	0	0,8084	0,1347	2,405
LK Freising	180007	3	0,8	3,59	3,484	289	229,6	1,26	1,728	1,04	0,36	2,85	2,032	0,5884	5,822
LK Freyung-Grafenau	78362	0	0,5	0	0,9758	21	92,3	0,23	0,3828	0	0,51	0	2,628	0,26	9,619
LK Fuerstenfeldbruck	219311	0	1,3	0	1,217	277	257,6	1,08	1,486	0	0,49	0	0,827	0,1777	2,137
LK Fuerth	117853	1	0,7	1,39	1,083	77	134,6	0,57	0,7903	1,3	0,53	2,43	1,4	0,2804	4,224
LK Garmisch-Partenkirchen	88424	3	0,6	5,22	4,646	100	101,6	0,98	1,358	3	0,57	5,3	3,453	0,672	10,5
LK Guenzburg	127027	0	0,7	0	1,217	135	154,5	0,87	1,209	0	0,44	0	1,015	0,1593	3,167
LK Hassberge	84384	0	0,5	0	0,5843	36	99,3	0,36	0,5185	0	0,49	0	1,153	0,1284	3,879
LK Hof	94801	0	0,6	0	0,7443	99	105,9	0,93	1,26	0	0,6	0	0,5988	0,1027	1,92
LK Kelheim	123058	0	0,6	0	1,221	175	150,3	1,16	1,592	0	0,43	0	0,7734	0,1598	2,082
LK Kitzingen	91155	1	0,5	1,92	1,289	76	108,5	0,7	0,9572	1,32	0,48	2,74	1,365	0,249	4,581
LK Kronach	66743	0	0,4	0	0,8694	17	75,7	0,22	0,3846	0	0,57	0	2,362	0,2971	8,597
LK Kulmbach	71566	0	0,5	0	0,6888	52	81,6	0,64	0,8683	0	0,56	0	0,8144	0,1215	2,635
LK Landsberg a.Lech	120302	0	0,7	0	1,214	91	143,7	0,63	0,8933	0	0,46	0	1,381	0,2442	4,01
LK Landshut	159895	1	0,8	1,19	2,077	166	193	0,86	1,197	0,6	0,44	1,38	1,756	0,4225	4,435
LK Lichtenfels	66776	0	0,4	0	0,6507	57	77,6	0,73	0,9803	0	0,52	0	0,6736	0,09045	2,324
LK Lindau	81981	0	0,5	0	0,7029	48	95,5	0,5	0,6955	0	0,52	0	1,022	0,08398	3,834
LK Main-Spessart	126158	1	0,8	1,28	1,034	54	145,1	0,37	0,5343	1,85	0,54	3,43	1,968	0,3833	6,327
LK Miesbach	100010	2	0,6	3,34	3,443	126	116,1	1,08	1,498	1,59	0,51	3,08	2,326	0,53	6,92

LK Miltenberg	128743	1	0,8	1,33	1,347	102	151,5	0,67	0,9267	0,98	0,5	1,98	1,467	0,2303	4,858
LK Muehldorf a.Inn	115872	0	0,6	0	1,398	133	139,9	0,95	1,312	0	0,45	0	1,08	0,2135	3,074
LK Muenchen	350473	1	1,9	0,51	1,577	489	419,6	1,17	1,61	0,2	0,46	0,44	0,9885	0,2792	2,402
LK Neu-Ulm	175204	0	1	0	1,279	280	211,4	1,32	1,82	0	0,46	0	0,706	0,09738	2,171
LK Neuburg-Schrobenhausen	97303	0	0,5	0	1,297	80	117,7	0,68	0,9537	0	0,44	0	1,381	0,2406	3,888
LK Neumarkt i.d.OPf.	134573	0	0,7	0	0,7192	143	163,6	0,87	1,188	0	0,44	0	0,6136	0,1155	1,945
LK Neustadt a.d.Aisch-Bad Windsheim	101014	1	0,6	1,73	1,232	34	119,5	0,28	0,4372	2,94	0,48	6,08	2,889	0,5875	9,77
LK Neustadt a.d.Waldnaab	94450	0	0,5	0	0,7333	43	111,7	0,39	0,5522	0	0,49	0	1,357	0,1995	4,475
LK Nuernberger Land	170792	0	1	0	0,6644	119	197	0,6	0,8316	0	0,52	0	0,8091	0,1381	2,228
LK Oberallgaeu	156008	0	0,9	0	0,8668	73	181,6	0,4	0,5782	0	0,52	0	1,519	0,2197	4,65
LK Ostallgaeu	141182	0	0,8	0	1,316	149	167,4	0,89	1,233	0	0,49	0	1,077	0,2216	3,004
LK Passau	192656	2	1,2	1,74	2,062	185	225,8	0,82	1,131	1,08	0,51	2,12	1,838	0,4832	4,71
LK Pfaffenhofen a.d.Ilm	128227	0	0,7	0	1,592	105	155,8	0,67	0,9542	0	0,42	0	1,687	0,333	4,804
LK Regen	77410	1	0,5	2,14	1,472	38	90,8	0,42	0,6115	2,63	0,51	5,12	2,453	0,3959	8,339
LK Regensburg	194070	1	1,1	0,95	1,233	165	231,6	0,71	0,9826	0,61	0,45	1,33	1,27	0,3112	3,521
LK Rhoen-Grabfeld	79635	0	0,5	0	0,4797	26	92,9	0,28	0,4119	0	0,52	0	1,197	0,1432	4,45
LK Rosenheim	261330	1	1,5	0,66	1,642	234	308,6	0,76	1,057	0,43	0,49	0,87	1,565	0,3702	4,035
LK Roth	126749	0	0,7	0	0,8771	60	149,1	0,4	0,5822	0	0,49	0	1,538	0,2697	4,432
LK Rottal-Inn	121502	3	0,7	4,26	3,98	108	145,2	0,74	1,042	2,78	0,49	5,72	3,872	1,065	10,97
LK Schwandorf	147872	0	0,8	0	0,6295	78	177,1	0,44	0,617	0	0,47	0	1,035	0,174	3,071
LK Schweinfurt	115445	0	0,7	0	0,4985	110	135,1	0,81	1,095	0	0,51	0	0,462	0,07074	1,506
LK Starnberg	136667	0	0,8	0	1,254	201	157,2	1,28	1,753	0	0,53	0	0,7248	0,128	2,091
LK Straubing-Bogen	101120	0	0,6	0	1,369	63	120,9	0,52	0,7538	0	0,46	0	1,852	0,339	5,313
LK Tirschenreuth	72046	1	0,4	2,27	1,358	43	83,9	0,51	0,7149	2,33	0,53	4,42	1,933	0,3021	6,87
LK Traunstein	177319	0	1,1	0	0,9879	124	207,8	0,6	0,8372	0	0,52	0	1,192	0,1902	3,331
LK Unterallgaeu	145341	4	0,8	4,81	4,041	146	174	0,84	1,158	2,74	0,48	5,73	3,519	0,9266	9,658
LK Weilheim-Schongau	135478	0	0,8	0	1,398	98	159,1	0,62	0,8706	0	0,5	0	1,625	0,303	4,7
LK Weissenburg-Gunzenhausen	94734	0	0,6	0	0,8164	108	111,3	0,97	1,311	0	0,5	0	0,6299	0,09436	1,96
LK Wuerzburg	162302	0	1	0	0,7746	192	190,5	1,01	1,374	0	0,5	0	0,5692	0,08036	1,664
LK Wunsiedel i.Fichtelgebirge	72655	3	0,5	6,1	3,314	60	81,2	0,74	1,003	5	0,61	8,26	3,361	0,6322	11,46

SK Amberg	42207	0	0,3	0	0,6285	23	49,9	0,46	0,6287	0	0,52	0	1,017	0,06451	3,836
SK Ansbach	41798	0	0,2	0	0,7683	32	50,6	0,63	0,8559	0	0,47	0	0,9105	0,0713	3,743
SK Aschaffenburg	71002	0	0,4	0	0,9053	47	85,6	0,55	0,7666	0	0,46	0	1,193	0,1329	4,272
SK Augsburg	296582	1	1,6	0,64	1,4	525	376,1	1,4	1,919	0,19	0,41	0,46	0,7352	0,1672	2,038
SK Bamberg	77373	0	0,4	0	0,5603	37	99,8	0,37	0,5212	0	0,41	0	1,083	0,06335	4,242
SK Bayreuth	74783	0	0,4	0	0,5137	33	99,7	0,33	0,4665	0	0,43	0	1,118	0,05877	4,436
SK Coburg	41072	2	0,3	7,92	4,895	39	49,3	0,79	1,114	5,13	0,51	10,01	4,449	0,6434	16,2
SK Erlangen	112528	1	0,6	1,81	1,64	98	149,6	0,65	0,9109	1,02	0,37	2,76	1,826	0,3343	5,679
SK Fuerth	128497	0	0,6	0	0,968	135	158,1	0,85	1,171	0	0,41	0	0,8351	0,1341	2,653
SK Hof	45825	1	0,3	3,52	2,53	37	54,1	0,68	0,9561	2,7	0,52	5,15	2,671	0,281	10,4
SK Ingolstadt	137392	1	0,7	1,42	1,951	276	171,1	1,61	2,205	0,36	0,41	0,88	0,8904	0,1647	2,664
SK Kaufbeuren	44398	4	0,3	14,99	13,84	69	52,4	1,32	1,828	5,8	0,51	11,39	7,66	1,379	21,88
SK Kempten	69151	0	0,4	0	1,085	73	85,1	0,86	1,163	0	0,48	0	0,9394	0,0633	3,435
SK Landshut	73411	0	0,4	0	2,117	140	90,5	1,55	2,11	0	0,44	0	1,009	0,09932	3,534
SK Memmingen	44100	1	0,3	3,92	4,599	77	52,7	1,46	2,001	1,3	0,48	2,68	2,318	0,3318	8,079
SK Muenchen	1484226	6	7,2	0,83	1,557	3723	1892,3	1,97	2,717	0,16	0,38	0,42	0,5767	0,2275	1,15
SK Nuernberg	518370	3	2,8	1,07	1,187	745	641	1,16	1,597	0,4	0,44	0,92	0,7493	0,2212	1,859
SK Passau	52803	0	0,3	0	1,701	76	69,1	1,1	1,503	0	0,43	0	1,143	0,1055	4,09
SK Regensburg	153094	0	0,7	0	1,084	172	203,9	0,84	1,16	0	0,36	0	0,9423	0,1047	3,672
SK Rosenheim	63551	0	0,3	0	2,558	180	78,8	2,29	3,104	0	0,44	0	0,8299	0,09687	2,993
SK Schwabach	40981	0	0,2	0	1,01	29	48,1	0,6	0,8373	0	0,49	0	1,229	0,1372	4,281
SK Schweinfurt	53426	0	0,3	0	0,9467	39	63,9	0,61	0,8322	0	0,51	0	1,142	0,1098	4,61
SK Straubing	47791	0	0,3	0	1,728	64	57,9	1,11	1,503	0	0,47	0	1,159	0,1308	4,491
SK Weiden i.d.OPf.	42743	0	0,3	0	1,272	48	51,1	0,94	1,262	0	0,5	0	1,011	0,09325	4,056
SK Wuerzburg	127934	1	0,7	1,46	2,267	226	172,7	1,31	1,797	0,44	0,4	1,11	1,266	0,2133	3,978

sMR=standardisierte Mortalitätsrate, Inf = Infektionen, sFR= standardisierte Fallrate, nFFR=naive Fallfatalitätsrate, eFFR=erwartete FFR, KI = Kredibilitätsintervall.

Z4.3. Daten für Oktober bis Dezember 2020 (Q4/20)

Landkreis	Population	Tode beob.	Tode erw.	sMR roh	sMR glatt	Inf beob.	Inf erw.	sFR roh	sFR glatt	nFFR	eFFR	sFFR roh	sFFR glatt	95% KI untere	95% KI obere
LK Aichach-Friedberg	134655	40	52,7	0,76	0,8456	2065	2161,6	0,96	1,01	1,94	2,44	0,79	0,8359	0,6096	1,108
LK Altoetting	111516	66	47,5	1,39	1,448	1943	1788,2	1,09	1,152	3,4	2,66	1,28	1,254	0,9436	1,604
LK Amberg-Sulzbach	103049	46	43	1,07	1,118	1440	1659,4	0,87	0,9192	3,19	2,59	1,23	1,214	0,8885	1,604
LK Ansbach	184591	53	73,2	0,72	0,8023	2333	2975,2	0,78	0,8291	2,27	2,46	0,92	0,966	0,7212	1,245
LK Aschaffenburg	174200	65	74,2	0,88	0,9977	2610	2791,9	0,93	0,9852	2,49	2,66	0,94	1,011	0,7681	1,287
LK Augsburg	253468	76	102,4	0,74	0,8497	4324	4063,8	1,06	1,122	1,76	2,52	0,7	0,7557	0,5862	0,9409
LK Bad Kissingen	103235	30	49,5	0,61	0,6366	937	1645,4	0,57	0,608	3,2	3,01	1,06	1,046	0,7217	1,443
LK Bad Toelz-Wolfratshausen	127917	19	53,6	0,35	0,4629	1685	2051,2	0,82	0,8663	1,13	2,61	0,43	0,5334	0,3584	0,7453
LK Bamberg	147163	29	56	0,52	0,5695	1482	2371,6	0,62	0,6641	1,96	2,36	0,83	0,8561	0,5983	1,157
LK Bayreuth	103664	13	45,7	0,28	0,3834	987	1657,4	0,6	0,6333	1,32	2,76	0,48	0,6042	0,3906	0,8714
LK Berchtesgadener Land	105929	42	48,7	0,86	0,9664	1982	1702,2	1,16	1,23	2,12	2,86	0,74	0,7842	0,5664	1,042
LK Cham	127998	39	54	0,72	0,7853	1672	2063	0,81	0,8596	2,33	2,62	0,89	0,9121	0,6577	1,215
LK Coburg	86747	64	39,4	1,63	1,725	1440	1385,5	1,04	1,096	4,44	2,84	1,56	1,571	1,188	2,027
LK Dachau	154899	54	56,7	0,95	1,084	2996	2502,1	1,2	1,262	1,8	2,27	0,8	0,8573	0,6417	1,106
LK Deggendorf	119478	41	48,3	0,85	0,9136	1772	1937,7	0,91	0,9704	2,31	2,49	0,93	0,9399	0,6753	1,25
LK Dillingen a.d.Donau	96562	51	38,5	1,33	1,309	1340	1556,3	0,86	0,918	3,81	2,47	1,54	1,424	1,037	1,87
LK Dingolfing-Landau	96683	51	36,5	1,4	1,376	1344	1566,9	0,86	0,9155	3,79	2,33	1,63	1,501	1,1	1,973
LK Donau-Ries	133783	46	52,8	0,87	0,9365	1856	2157,5	0,86	0,9105	2,48	2,45	1,01	1,027	0,7526	1,34
LK Ebersberg	143649	71	52,7	1,35	1,398	2401	2311,8	1,04	1,101	2,96	2,28	1,3	1,267	0,9612	1,608
LK Eichstaett	132881	34	46,5	0,73	0,7967	1716	2148,2	0,8	0,8458	1,98	2,17	0,91	0,9403	0,6735	1,266
LK Erding	138182	49	46,9	1,05	1,179	2695	2241,6	1,2	1,267	1,82	2,09	0,87	0,9291	0,6832	1,205
LK Erlangen-Hoechstadt	137262	55	55,5	0,99	1,061	1825	2193,1	0,83	0,8809	3,01	2,53	1,19	1,203	0,8941	1,551
LK Forchheim	116203	43	46,9	0,92	0,9827	1502	1867,5	0,8	0,851	2,86	2,51	1,14	1,153	0,8435	1,523
LK Freising	180007	17	57,1	0,3	0,4409	2501	2952,2	0,85	0,8926	0,68	1,93	0,35	0,4929	0,3281	0,6903
LK Freyung-Grafenau	78362	55	34	1,62	1,8	1904	1263	1,51	1,588	2,89	2,69	1,07	1,132	0,8528	1,466

LK Fuerstenfeldbruck	219311	42	92,3	0,46	0,575	3738	3509,8	1,07	1,121	1,12	2,63	0,43	0,5117	0,3764	0,6684
LK Fuerth	117853	76	52,7	1,44	1,525	1813	1877,7	0,97	1,022	4,19	2,81	1,49	1,489	1,136	1,88
LK Garmisch-Partenkirchen	88424	24	42,9	0,56	0,615	1113	1414,4	0,79	0,8342	2,16	3,03	0,71	0,7359	0,5012	1,023
LK Guenzburg	127027	68	48,6	1,4	1,56	2801	2054,7	1,36	1,435	2,43	2,36	1,03	1,085	0,8288	1,373
LK Hassberge	84384	36	35,1	1,03	1,139	1273	1355,7	0,94	0,9882	2,83	2,59	1,09	1,151	0,8179	1,554
LK Hof	94801	38	47,6	0,8	0,9223	1432	1508,4	0,95	0,999	2,65	3,15	0,84	0,9215	0,6661	1,221
LK Kelheim	123058	17	45,4	0,37	0,4465	1267	1991,1	0,64	0,6764	1,34	2,28	0,59	0,659	0,4306	0,9407
LK Kitzingen	91155	21	37,4	0,56	0,6405	976	1468,5	0,66	0,7038	2,15	2,54	0,85	0,9084	0,608	1,278
LK Kronach	66743	28	31,7	0,88	1,042	1077	1068,4	1,01	1,058	2,6	2,97	0,88	0,9836	0,6863	1,333
LK Kulmbach	71566	16	33,7	0,47	0,5227	694	1145,3	0,61	0,6488	2,31	2,94	0,78	0,8044	0,5171	1,171
LK Landsberg a.Lech	120302	23	47,4	0,49	0,5765	1624	1934	0,84	0,8878	1,42	2,45	0,58	0,6481	0,4452	0,8948
LK Landshut	159895	82	59,4	1,38	1,435	2455	2578,2	0,95	1,01	3,34	2,3	1,45	1,419	1,091	1,783
LK Lichtenfels	66776	23	29,7	0,77	0,9127	963	1072,2	0,9	0,9432	2,39	2,77	0,86	0,966	0,6569	1,335
LK Lindau	81981	28	36,2	0,77	0,8636	1270	1312,9	0,97	1,021	2,2	2,76	0,8	0,8445	0,5811	1,176
LK Main-Spessart	126158	152	57,3	2,65	2,744	1836	2018,2	0,91	0,9673	8,28	2,84	2,92	2,832	2,276	3,429
LK Miesbach	100010	15	43,9	0,34	0,4721	1375	1599,9	0,86	0,9065	1,09	2,74	0,4	0,5198	0,3378	0,7436
LK Miltenberg	128743	46	54,3	0,85	0,9769	1881	2067,6	0,91	0,9581	2,45	2,63	0,93	1,018	0,7523	1,334
LK Muehldorf a.Inn	115872	53	45,4	1,17	1,305	2343	1870,5	1,25	1,32	2,26	2,43	0,93	0,9864	0,7381	1,268
LK Muenchen	350473	82	139,9	0,59	0,6587	5224	5628,5	0,93	0,9804	1,57	2,49	0,63	0,6705	0,5199	0,8344
LK Neu-Ulm	175204	48	69,5	0,69	0,777	2655	2828,8	0,94	0,9918	1,81	2,46	0,74	0,7819	0,5689	1,025
LK Neuburg-Schrobenhausen	97303	33	36,8	0,9	0,9665	1421	1569,8	0,91	0,9576	2,32	2,34	0,99	1,008	0,716	1,352
LK Neumarkt i.d.OPf.	134573	62	51	1,22	1,268	1912	2178,1	0,88	0,9306	3,24	2,34	1,39	1,36	1,021	1,75
LK Neustadt a.d.Aisch-Bad Windsheim	101014	29	41,6	0,7	0,8021	1295	1623,3	0,8	0,8411	2,24	2,56	0,87	0,9521	0,6718	1,289
LK Neustadt a.d.Waldnaab	94450	25	39,3	0,64	0,806	1542	1520,5	1,01	1,064	1,62	2,58	0,63	0,756	0,5307	1,029
LK Nuernberger Land	170792	72	74,6	0,97	1,042	2329	2728,4	0,85	0,9027	3,09	2,73	1,13	1,153	0,8846	1,45
LK Oberallgaeu	156008	39	69,2	0,56	0,6408	2101	2497,6	0,84	0,8885	1,86	2,77	0,67	0,72	0,5222	0,9535
LK Ostallgaeu	141182	42	58,7	0,72	0,7937	2083	2264,2	0,92	0,9716	2,02	2,59	0,78	0,8153	0,5962	1,072
LK Passau	192656	141	83,9	1,68	1,866	4832	3095,8	1,56	1,646	2,92	2,71	1,08	1,131	0,9111	1,367
LK Pfaffenhofen a.d.Ilm	128227	85	46,3	1,83	1,856	2063	2072,4	1	1,057	4,12	2,24	1,84	1,753	1,344	2,206
LK Regen	77410	77	34,1	2,26	2,521	2190	1245,6	1,76	1,845	3,52	2,73	1,29	1,364	1,054	1,724

LK Regensburg	194070	87	74,9	1,16	1,216	2644	3123,3	0,85	0,8977	3,29	2,4	1,37	1,352	1,044	1,697
LK Rhoen-Grabfeld	79635	16	35	0,46	0,592	912	1275,4	0,72	0,7534	1,75	2,74	0,64	0,7845	0,5154	1,129
LK Rosenheim	261330	156	109,6	1,42	1,537	5180	4193,2	1,24	1,305	3,01	2,61	1,15	1,175	0,9454	1,416
LK Roth	126749	52	52,2	1	1,078	1810	2034,7	0,89	0,941	2,87	2,57	1,12	1,143	0,85	1,481
LK Rottal-Inn	121502	42	51	0,82	0,9892	2349	1960	1,2	1,26	1,79	2,6	0,69	0,7834	0,5757	1,023
LK Schwandorf	147872	52	59,9	0,87	1,002	2503	2389,1	1,05	1,103	2,08	2,51	0,83	0,9072	0,6728	1,175
LK Schweinfurt	115445	57	49,6	1,15	1,271	1757	1851,3	0,95	0,9982	3,24	2,68	1,21	1,271	0,9625	1,635
LK Starnberg	136667	17	61	0,28	0,3571	1495	2172,2	0,69	0,7293	1,14	2,81	0,41	0,4887	0,3199	0,6938
LK Straubing-Bogen	101120	55	39,3	1,4	1,425	1545	1628,9	0,95	1,007	3,56	2,41	1,48	1,412	1,051	1,827
LK Tirschenreuth	72046	41	32,3	1,27	1,29	1006	1157,4	0,87	0,9228	4,08	2,79	1,46	1,396	0,9949	1,862
LK Traunstein	177319	69	78,7	0,88	1,011	3711	2844	1,3	1,375	1,86	2,77	0,67	0,7337	0,5572	0,9255
LK Unterallgaeu	145341	45	59,9	0,75	0,795	1921	2339,1	0,82	0,8714	2,34	2,56	0,91	0,9106	0,6634	1,207
LK Weilheim-Schongau	135478	24	57,7	0,42	0,503	1745	2168,7	0,8	0,8497	1,38	2,66	0,52	0,5909	0,4075	0,8116
LK Weissenburg-Gunzenhausen	94734	35	40,5	0,86	0,8942	1139	1519,3	0,75	0,7972	3,07	2,66	1,15	1,12	0,7933	1,509
LK Wuerzburg	162302	32	68,6	0,47	0,5038	1409	2602,5	0,54	0,5765	2,27	2,64	0,86	0,8725	0,6107	1,193
LK Wunsiedel i.Fichtelgebirge	72655	27	37	0,73	0,827	960	1155,7	0,83	0,8771	2,81	3,2	0,88	0,9412	0,65	1,297
SK Amberg	42207	4	19,2	0,21	0,4274	471	680,9	0,69	0,7279	0,85	2,82	0,3	0,5863	0,3198	0,9501
SK Ansbach	41798	6	17,3	0,35	0,5063	442	676,2	0,65	0,6911	1,36	2,56	0,53	0,732	0,4137	1,175
SK Aschaffenburg	71002	40	28,9	1,39	1,425	1011	1150,6	0,88	0,932	3,96	2,51	1,58	1,527	1,091	2,046
SK Augsburg	296582	202	112,1	1,8	1,977	7538	4881,8	1,54	1,63	2,68	2,3	1,17	1,211	0,9849	1,442
SK Bamberg	77373	50	29,1	1,72	1,727	1044	1283,2	0,81	0,8644	4,79	2,27	2,11	1,995	1,441	2,642
SK Bayreuth	74783	20	31	0,64	0,6861	786	1254,1	0,63	0,6656	2,54	2,47	1,03	1,03	0,6539	1,49
SK Coburg	41072	25	18,7	1,34	1,336	552	666,9	0,83	0,8804	4,53	2,81	1,61	1,517	1,014	2,156
SK Erlangen	112528	37	38,9	0,95	0,9862	1458	1874,8	0,78	0,8266	2,54	2,07	1,22	1,191	0,8427	1,605
SK Fuerth	128497	48	46,3	1,04	1,196	2417	2094,4	1,15	1,215	1,99	2,21	0,9	0,9825	0,7227	1,278
SK Hof	45825	36	21,3	1,69	1,799	906	737,6	1,23	1,295	3,97	2,88	1,38	1,388	0,975	1,888
SK Ingolstadt	137392	22	49,8	0,44	0,5844	1996	2240,2	0,89	0,9381	1,1	2,22	0,5	0,6217	0,4277	0,8573
SK Kaufbeuren	44398	32	19,6	1,64	1,659	829	713,1	1,16	1,23	3,86	2,74	1,41	1,348	0,9242	1,872
SK Kempten	69151	19	29,6	0,64	0,7134	926	1126,3	0,82	0,8698	2,05	2,63	0,78	0,8189	0,5341	1,183
SK Landshut	73411	38	29,2	1,3	1,359	1143	1197,5	0,95	1,011	3,32	2,44	1,36	1,342	0,9498	1,817

SK Memmingen	44100	13	18,6	0,7	0,7864	611	710,9	0,86	0,909	2,13	2,62	0,81	0,8643	0,5288	1,299
SK Muenchen	1484226	546	508,8	1,07	1,177	28515	24460,8	1,17	1,231	1,91	2,08	0,92	0,954	0,8017	1,096
SK Nuernberg	518370	397	202,5	1,96	2,16	11748	8458,3	1,39	1,465	3,38	2,39	1,41	1,471	1,234	1,698
SK Passau	52803	60	21,8	2,75	2,864	1374	881,3	1,56	1,65	4,37	2,47	1,77	1,733	1,284	2,249
SK Regensburg	153094	42	51,6	0,81	0,911	2344	2563,8	0,91	0,9651	1,79	2,01	0,89	0,9421	0,6748	1,254
SK Rosenheim	63551	18	24,8	0,72	0,9265	1391	1036	1,34	1,412	1,29	2,4	0,54	0,6553	0,4259	0,9356
SK Schwabach	40981	57	17,3	3,29	3,24	817	656,8	1,24	1,321	6,98	2,64	2,64	2,45	1,794	3,227
SK Schweinfurt	53426	18	24,5	0,73	0,9167	872	861,7	1,01	1,062	2,06	2,84	0,73	0,8624	0,5656	1,237
SK Straubing	47791	9	20	0,45	0,6224	630	777,5	0,81	0,8549	1,43	2,57	0,56	0,7272	0,4348	1,124
SK Weiden i.d.OPf.	42743	17	19,2	0,89	1,08	834	691,9	1,21	1,266	2,04	2,77	0,74	0,8524	0,5447	1,242
SK Wuerzburg	127934	18	49,7	0,36	0,4516	1296	2160,7	0,6	0,6331	1,39	2,3	0,6	0,7123	0,4621	1,023

sMR=standardisierte Mortalitätsrate, Inf = Infektionen, sFR= standardisierte Fallrate, nFFR=naive Fallfatalitätsrate, eFFR=erwartete FFR, KI = Kredibilitätsintervall.

Z4.4. Daten für Januar bis März 2021 (Q1/21)

**Landkreis**	**Population**	**Tode beob.**	**Tode erw.**	**sMR roh**	**sMR glatt**	**Inf beob.**	**Inf erw.**	**sIR roh**	**sIR glatt**	**nIFR**	**eIFR**	**sIFR roh**	**sIFR glatt**	**95% KI untere**	**95% KI obere**
LK Aichach-Friedberg	134655	29	51,6	0,56	0,5256	1087	1860,1	0,58	0,555	2,67	2,77	0,96	0,9415	0,6536	1,285
LK Altoetting	111516	70	46,4	1,51	1,348	1531	1544	0,99	0,9396	4,57	3,01	1,52	1,427	1,058	1,838
LK Amberg-Sulzbach	103049	37	42,1	0,88	0,8542	1413	1433,1	0,99	0,9313	2,62	2,94	0,89	0,9121	0,6514	1,224
LK Ansbach	184591	69	71,7	0,96	0,9516	2272	2560,8	0,89	0,8347	3,04	2,8	1,08	1,134	0,8591	1,435
LK Aschaffenburg	174200	75	72,6	1,03	1,032	2699	2414,3	1,12	1,051	2,78	3,01	0,92	0,9763	0,7366	1,243
LK Augsburg	253468	89	100,2	0,89	0,842	2703	3502,2	0,77	0,7274	3,29	2,86	1,15	1,151	0,8849	1,448
LK Bad Kissingen	103235	36	48,3	0,74	0,7534	1225	1430,8	0,86	0,8046	2,94	3,38	0,87	0,9312	0,662	1,254
LK Bad Toelz-Wolfratshausen	127917	35	52,4	0,67	0,6683	1369	1770,1	0,77	0,7277	2,56	2,96	0,86	0,9131	0,6515	1,223
LK Bamberg	147163	52	55	0,95	0,9091	1766	2040,9	0,87	0,8173	2,94	2,69	1,09	1,106	0,8152	1,436
LK Bayreuth	103664	101	44,7	2,26	2,131	2285	1436,4	1,59	1,498	4,42	3,11	1,42	1,414	1,102	1,772
LK Berchtesgadener Land	105929	31	47,5	0,65	0,7103	2266	1474,5	1,54	1,442	1,37	3,22	0,42	0,4898	0,3392	0,6741

LK Cham	127998	80	52,9	1,51	1,458	2386	1781,7	1,34	1,26	3,35	2,97	1,13	1,15	0,8712	1,459
LK Coburg	86747	46	38,5	1,2	1,225	1542	1202,6	1,28	1,203	2,98	3,2	0,93	1,013	0,7488	1,33
LK Dachau	154899	41	55,7	0,74	0,7415	1848	2147,9	0,86	0,8084	2,22	2,59	0,86	0,9113	0,6547	1,209
LK Deggendorf	119478	66	47,2	1,4	1,366	2431	1669,1	1,46	1,37	2,71	2,83	0,96	0,9906	0,7418	1,273
LK Dillingen a.d.Donau	96562	22	37,7	0,58	0,5824	907	1339,5	0,68	0,6384	2,43	2,81	0,86	0,9081	0,6121	1,274
LK Dingolfing-Landau	96683	17	35,8	0,47	0,5977	1362	1346,2	1,01	0,9496	1,25	2,66	0,47	0,6263	0,42	0,8777
LK Donau-Ries	133783	71	51,8	1,37	1,217	1495	1856,1	0,81	0,7625	4,75	2,79	1,7	1,586	1,195	2,046
LK Ebersberg	143649	87	51,7	1,68	1,51	1857	1983,6	0,94	0,8859	4,68	2,6	1,8	1,694	1,28	2,147
LK Eichstaett	132881	18	45,7	0,39	0,4904	1345	1838,1	0,73	0,6869	1,34	2,49	0,54	0,7102	0,476	0,9789
LK Erding	138182	44	46,1	0,95	0,8902	1617	1918,8	0,84	0,7956	2,72	2,4	1,13	1,113	0,8054	1,472
LK Erlangen-Hoechstadt	137262	47	54,4	0,86	0,8608	1646	1891,7	0,87	0,8197	2,86	2,87	0,99	1,045	0,7694	1,361
LK Forchheim	116203	54	46	1,17	1,159	1789	1610,9	1,11	1,044	3,02	2,85	1,06	1,103	0,815	1,441
LK Freising	180007	54	56,2	0,96	0,9136	2222	2517,4	0,88	0,8314	2,43	2,23	1,09	1,092	0,8089	1,427
LK Freyung-Grafenau	78362	43	33,2	1,29	1,302	1674	1092,8	1,53	1,441	2,57	3,04	0,84	0,8993	0,6526	1,192
LK Fuerstenfeldbruck	219311	35	90,2	0,39	0,447	2249	3029	0,74	0,6962	1,56	2,98	0,52	0,6382	0,4554	0,852
LK Fuerth	117853	53	51,5	1,03	1,035	1592	1628,4	0,98	0,9193	3,33	3,16	1,05	1,12	0,8313	1,448
LK Garmisch-Partenkirchen	88424	36	41,8	0,86	0,842	1131	1229,3	0,92	0,8636	3,18	3,4	0,94	0,9696	0,687	1,306
LK Guenzburg	127027	23	47,6	0,48	0,5149	1327	1765	0,75	0,7062	1,73	2,7	0,64	0,7255	0,4987	0,9972
LK Hassberge	84384	31	34,4	0,9	0,8991	1091	1170,6	0,93	0,877	2,84	2,93	0,97	1,02	0,7168	1,378
LK Hof	94801	48	46,4	1,04	1,142	2287	1315,7	1,74	1,63	2,1	3,52	0,6	0,6972	0,5131	0,9046
LK Kelheim	123058	39	44,5	0,88	0,8175	1379	1708	0,81	0,7629	2,83	2,61	1,08	1,066	0,7767	1,422
LK Kitzingen	91155	50	36,6	1,37	1,207	994	1266,4	0,78	0,744	5,03	2,89	1,74	1,614	1,169	2,15
LK Kronach	66743	49	30,9	1,58	1,498	1289	930,2	1,39	1,308	3,8	3,33	1,14	1,14	0,8315	1,499
LK Kulmbach	71566	72	32,9	2,19	2,129	1870	996	1,88	1,764	3,85	3,3	1,17	1,201	0,9163	1,532
LK Landsberg a.Lech	120302	28	46,4	0,6	0,5336	924	1664,4	0,56	0,5281	3,03	2,79	1,09	1,005	0,6839	1,379
LK Landshut	159895	38	58,3	0,65	0,7024	2115	2214	0,96	0,8974	1,8	2,63	0,68	0,7784	0,5639	1,024
LK Lichtenfels	66776	27	29	0,93	0,9823	1068	929,2	1,15	1,082	2,53	3,13	0,81	0,9036	0,6316	1,219
LK Lindau	81981	22	35,3	0,62	0,5777	954	1136	0,84	0,7913	2,31	3,11	0,74	0,7263	0,4684	1,054
LK Main-Spessart	126158	35	56	0,63	0,6485	1408	1750,6	0,8	0,7562	2,49	3,2	0,78	0,853	0,6081	1,137
LK Miesbach	100010	33	42,8	0,77	0,7644	1240	1384,6	0,9	0,8424	2,66	3,09	0,86	0,9023	0,6376	1,227

LK Miltenberg	128743	55	53,2	1,03	1,055	2128	1785,9	1,19	1,119	2,58	2,98	0,87	0,9378	0,6963	1,221
LK Muehldorf a.Inn	115872	54	44,5	1,21	1,191	1941	1608,5	1,21	1,133	2,78	2,77	1,01	1,045	0,7777	1,364
LK Muenchen	350473	92	136,9	0,67	0,6824	4206	4841,5	0,87	0,8164	2,19	2,83	0,77	0,831	0,6463	1,038
LK Neu-Ulm	175204	25	68	0,37	0,3869	1588	2434	0,65	0,6141	1,57	2,8	0,56	0,6272	0,4244	0,8719
LK Neuburg-Schrobenhausen	97303	14	36,1	0,39	0,5007	1006	1348,4	0,75	0,6985	1,39	2,68	0,52	0,7126	0,4633	1,018
LK Neumarkt i.d.OPf.	134573	31	50	0,62	0,6988	1837	1871,2	0,98	0,9204	1,69	2,67	0,63	0,755	0,5374	1,003
LK Neustadt a.d.Aisch-Bad Windsheim	101014	48	40,7	1,18	1,144	1353	1400,3	0,97	0,9078	3,55	2,91	1,22	1,253	0,9181	1,644
LK Neustadt a.d.Waldnaab	94450	51	38,4	1,33	1,331	1975	1312,8	1,5	1,415	2,58	2,93	0,88	0,9354	0,6864	1,219
LK Nuernberger Land	170792	40	72,9	0,55	0,597	2021	2362,6	0,86	0,8049	1,98	3,09	0,64	0,7377	0,5316	0,9653
LK Oberallgaeu	156008	12	67,6	0,18	0,2808	1668	2161,7	0,77	0,7217	0,72	3,13	0,23	0,3871	0,2527	0,5538
LK Ostallgaeu	141182	28	57,4	0,49	0,5114	1469	1951,6	0,75	0,7069	1,91	2,94	0,65	0,7191	0,5011	0,9739
LK Passau	192656	167	82	2,04	1,988	5001	2678,3	1,87	1,756	3,34	3,06	1,09	1,126	0,897	1,368
LK Pfaffenhofen a.d.Ilm	128227	42	45,5	0,92	0,8566	1447	1778	0,81	0,7676	2,9	2,56	1,13	1,109	0,806	1,478
LK Regen	77410	63	33,3	1,89	1,881	2028	1078	1,88	1,763	3,11	3,09	1,01	1,061	0,8025	1,351
LK Regensburg	194070	66	73,4	0,9	0,8094	2006	2688	0,75	0,7079	3,29	2,73	1,2	1,137	0,851	1,478
LK Rhoen-Grabfeld	79635	25	34,2	0,73	0,7696	993	1103,3	0,9	0,8442	2,52	3,1	0,81	0,9067	0,6167	1,256
LK Rosenheim	261330	78	107,2	0,73	0,7285	3529	3618,4	0,98	0,9175	2,21	2,96	0,75	0,7894	0,6038	0,9944
LK Roth	126749	97	51,1	1,9	1,755	1923	1756,4	1,09	1,033	5,04	2,91	1,73	1,688	1,305	2,122
LK Rottal-Inn	121502	66	49,9	1,32	1,299	2283	1690,6	1,35	1,269	2,89	2,95	0,98	1,018	0,7741	1,296
LK Schwandorf	147872	60	58,6	1,02	1,065	2793	2059,4	1,36	1,272	2,15	2,85	0,75	0,832	0,6249	1,067
LK Schweinfurt	115445	29	48,5	0,6	0,605	1075	1600,2	0,67	0,6343	2,7	3,03	0,89	0,9485	0,6596	1,288
LK Starnberg	136667	72	59,5	1,21	1,073	1385	1879,8	0,74	0,6986	5,2	3,17	1,64	1,527	1,144	1,962
LK Straubing-Bogen	101120	39	38,5	1,01	1,01	1582	1402,1	1,13	1,061	2,47	2,75	0,9	0,9463	0,6877	1,254
LK Tirschenreuth	72046	66	31,6	2,09	2,164	2508	1002,9	2,5	2,344	2,63	3,15	0,84	0,9181	0,6942	1,175
LK Traunstein	177319	39	76,9	0,51	0,5779	2816	2460	1,14	1,074	1,38	3,13	0,44	0,5351	0,3855	0,7084
LK Unterallgaeu	145341	56	58,6	0,96	0,9089	2073	2014,6	1,03	0,9663	2,7	2,91	0,93	0,9348	0,6987	1,224
LK Weilheim-Schongau	135478	32	56,4	0,57	0,5483	1195	1872,3	0,64	0,6025	2,68	3,01	0,89	0,9052	0,641	1,222
LK Weissenburg-Gunzenhausen	94734	46	39,6	1,16	1,074	1096	1312,2	0,84	0,7896	4,2	3,02	1,39	1,354	0,9827	1,792
LK Wuerzburg	162302	24	67,1	0,36	0,3857	1156	2248	0,51	0,4858	2,08	2,99	0,7	0,7902	0,5409	1,083
LK Wunsiedel i.Fichtelgebirge	72655	110	36	3,05	2,953	2478	1008,3	2,46	2,31	4,44	3,57	1,24	1,271	0,9913	1,579

SK Amberg	42207	10	18,8	0,53	0,6391	567	589,6	0,96	0,9023	1,76	3,18	0,55	0,7053	0,414	1,093
SK Ansbach	41798	26	16,9	1,54	1,472	673	582,7	1,16	1,086	3,86	2,91	1,33	1,35	0,9164	1,895
SK Aschaffenburg	71002	50	28,2	1,77	1,627	1172	991,9	1,18	1,115	4,27	2,84	1,5	1,451	1,059	1,924
SK Augsburg	296582	135	109,7	1,23	1,193	4122	4186,1	0,98	0,9263	3,28	2,62	1,25	1,28	1,01	1,577
SK Bamberg	77373	19	28,5	0,67	0,6802	822	1099,8	0,75	0,7039	2,31	2,59	0,89	0,9613	0,612	1,403
SK Bayreuth	74783	69	30,2	2,28	2,149	1447	1074,5	1,35	1,27	4,77	2,82	1,69	1,683	1,252	2,175
SK Coburg	41072	57	18,3	3,12	2,912	985	577,2	1,71	1,608	5,79	3,16	1,83	1,802	1,316	2,363
SK Erlangen	112528	34	38,1	0,89	0,8117	1022	1597,7	0,64	0,6085	3,33	2,39	1,39	1,327	0,906	1,81
SK Fuerth	128497	98	45,4	2,16	2,047	2177	1798,4	1,21	1,14	4,5	2,53	1,78	1,786	1,376	2,248
SK Hof	45825	47	20,7	2,27	2,231	1486	638,7	2,33	2,186	3,16	3,24	0,97	1,015	0,7245	1,361
SK Ingolstadt	137392	22	48,8	0,45	0,4979	1396	1919,3	0,73	0,6834	1,58	2,54	0,62	0,7245	0,4923	1,01
SK Kaufbeuren	44398	7	19,1	0,37	0,4457	455	616,2	0,74	0,6926	1,54	3,1	0,5	0,641	0,3559	1,033
SK Kempten	69151	5	28,9	0,17	0,2337	552	970,1	0,57	0,5359	0,91	2,98	0,3	0,4336	0,2097	0,7485
SK Landshut	73411	51	28,5	1,79	1,678	1306	1030	1,27	1,195	3,91	2,77	1,41	1,397	1,012	1,854
SK Memmingen	44100	7	18,2	0,39	0,4872	529	613,2	0,86	0,8085	1,32	2,96	0,45	0,6003	0,3336	0,9526
SK Muenchen	1484226	342	499,4	0,68	0,6734	17321	20930	0,83	0,7786	1,97	2,39	0,83	0,8594	0,6968	1,016
SK Nuernberg	518370	396	198,1	2	1,973	10916	7270	1,5	1,411	3,63	2,73	1,33	1,389	1,145	1,642
SK Passau	52803	31	21,3	1,46	1,46	1292	756,3	1,71	1,607	2,4	2,81	0,85	0,9043	0,617	1,238
SK Regensburg	153094	15	50,6	0,3	0,3614	1503	2187,4	0,69	0,6458	1	2,31	0,43	0,5564	0,3517	0,8162
SK Rosenheim	63551	14	24,3	0,58	0,6727	1034	889,7	1,16	1,089	1,35	2,73	0,5	0,614	0,3765	0,9131
SK Schwabach	40981	39	16,9	2,3	2,047	657	567,4	1,16	1,096	5,94	2,99	1,99	1,859	1,302	2,535
SK Schweinfurt	53426	25	23,9	1,05	0,9304	491	744,6	0,66	0,6251	5,09	3,2	1,59	1,482	0,962	2,128
SK Straubing	47791	18	19,6	0,92	0,8257	515	670,8	0,77	0,729	3,5	2,92	1,2	1,128	0,7061	1,669
SK Weiden i.d.OPf.	42743	34	18,7	1,82	1,781	1083	598,5	1,81	1,7	3,14	3,12	1,01	1,042	0,7176	1,425
SK Wuerzburg	127934	31	48,6	0,64	0,6238	1122	1848,7	0,61	0,5714	2,76	2,63	1,05	1,086	0,7373	1,516

sMR=standardisierte Mortalitätsrate, Inf = Infektionen, sFR= standardisierte Fallrate, nFFR=naive Fallfatalitätsrate, eFFR=erwartete FFR, KI = Kredibilitätsintervall.
